# Integrative analysis of structural variations using short-reads and linked-reads yields highly specific and sensitive predictions

**DOI:** 10.1371/journal.pcbi.1008397

**Published:** 2020-11-23

**Authors:** Riccha Sethi, Julia Becker, Jos de Graaf, Martin Löwer, Martin Suchan, Ugur Sahin, David Weber

**Affiliations:** 1 TRON—Translational Oncology at the University Medical Center of Johannes Gutenberg University Mainz gGmbH, Mainz, Germany; 2 University Medical Center of the Johannes Gutenberg University, Mainz, Germany; University of Cambridge, UNITED KINGDOM

## Abstract

Genetic diseases are driven by aberrations of the human genome. Identification of such aberrations including structural variations (SVs) is key to our understanding. Conventional short-reads whole genome sequencing (cWGS) can identify SVs to base-pair resolution, but utilizes only short-range information and suffers from high false discovery rate (FDR). Linked-reads sequencing (10XWGS) utilizes long-range information by linkage of short-reads originating from the same large DNA molecule. This can mitigate alignment-based artefacts especially in repetitive regions and should enable better prediction of SVs. However, an unbiased evaluation of this technology is not available. In this study, we performed a comprehensive analysis of different types and sizes of SVs predicted by both the technologies and validated with an independent PCR based approach. The SVs commonly identified by both the technologies were highly specific, while validation rate dropped for uncommon events. A particularly high FDR was observed for SVs only found by 10XWGS. To improve FDR and sensitivity, statistical models for both the technologies were trained. Using our approach, we characterized SVs from the MCF7 cell line and a primary breast cancer tumor with high precision. This approach improves SV prediction and can therefore help in understanding the underlying genetics in various diseases.

## Author summary

Cancer and many other diseases are often driven by structural rearrangements in the patients. Their precise identification is necessary to understand evolution and cure for the disease. In this study, we have compared two sequencing technologies for the identification of structural variations i.e. Illumina’s short-reads and 10X Genomics linked-reads sequencing. Short-reads sequencing is already known to have high false discovery rate for structural variations, while, an unbiased performance evaluation of linked-reads sequencing is missing. Hence, we evaluate the performance of these two technologies using computational and PCR based methodologies. Moreover, we also present a statistical approach to increase their performance, supporting better detection of structural variations and thus further research into disease biology.

This is a *PLOS Computational Biology* Benchmarking paper.

## Introduction

Structural variations (SVs) are large rearrangements in the genome, including deletions, duplications, inversions, translocations and insertions, and drive the development of diseases like cancer, autism and mendelian disorders [1]. One well-known example is the Philadelphia chromosome, an interchromosomal rearrangement (translocation) between chromosome 22 and chromosome 9 in chronic myeloid leukemia. This SV causes the fusion of two distantly located genes, BCR and ABL1, forming an active tyrosine kinase which leads to uncontrolled growth of cells [2]. Even a single SV can alter the expression of genes by functional impacts such as enhancer hijacking, truncation or disruption of tumor suppressor genes and amplifications of oncogenes. Hence, resolving such chromosomal rearrangements holds the key to understanding the causes behind genetic diseases [1].

Historically, large genomic alterations could be identified microscopically using karyotyping that allows genome wide identification but only at a very low resolution. More recently, SVs that lead to copy number variations (CNVs) could also be identified using array-comparative genomic hybridization, but without breakpoint information.

The onset of next-generation sequencing enabled a genome-wide read out for all SV types at base pair resolution. In theory, conventional whole genome sequencing (cWGS) by Illumina allows the identification of all SVs in an individual sample. However, a major shortcoming of this technology is contributed by the short-fragment DNA library preparation for sequencing with DNA fragment of size typically below 0.5 kb. Moreover, these short-fragments are sequenced with even shorter reads of length typically 2x150 bp. Therefore, this technique proves inefficient in aligning reads originating from repetitive elements in the human genome that are often associated with SVs [3]. Multiple tools and algorithms exist for prediction of SVs from cWGS data [4], but due to the described limitations, they often lack sensitivity and have high false discovery rates (FDR), especially in repetitive regions [5]. To reduce FDR, many studies consider SVs predicted by multiple bioinformatics tools in consensus as true positives [6–8] at the cost of losing sensitivity. This approach might not be appropriate in a clinical setting where the treatment of a patient relies on sensitive discovery of true somatic variants. In general, these bioinformatics tools identify SVs by using up to three different signals from aligned reads: (a) Read-depth information for inferring CNVs from non-uniform coverage in the regions, (b) discordant read-pairs that map with unexpected distance or orientation, and (c) split reads that have portions of a read mapping to different locations.

To deal with limitations of cWGS, recently “linked-reads sequencing” (10XWGS) technology was introduced. This utilizes reads derived from high molecular weight (HMW) DNA with typical fragment size between 50–100 kb in order to supply long-range information [7]. This approach captures HMW DNA molecules in so-called “Gel beads in Emulsion (GEM)”. After encapsulation, HMW DNA is sheared into smaller fragments (0.5 kb), labelled with GEM specific barcodes and subjected to cWGS (2x150 bp). The attached barcodes link each short read-pair to its originating HMW DNA. The 10XWGS bioinformatics pipeline (Long Ranger) utilizes this information to reconstruct the initial long HMW DNA molecule. This also allows linking longer sections of the genome together into a phased haplotype and resolving SVs in low complexity regions of the genome. Theoretically, this should enable highly specific and sensitive prediction of SVs.

Several studies have recently used 10XWGS for molecular characterization of either large-sized SVs [8,9] or complex genomic rearrangements [10]. This is not limited to the normal human genome [11] but also feasible for different types of cancer and other diseases [12–14]. However, these studies predominantly use 10XWGS technology for orthogonal validation of SVs, but a comprehensive comparison of all SVs identified with 10XWGS and cWGS as an independent finding is currently not available.

Here, we performed an in-depth analysis of SVs from the MCF7 breast cancer cell line and a primary breast cancer sample. The goals of this study were: a) to evaluate and compare 10XWGS and cWGS technology for the prediction of different types and sizes of SVs; b) to identify an approach to predict highly specific SVs from both the technologies; c) to analyse GEM count as a predictor of true positive SVs. With this analysis, we also propose a statistical approach to determine highly specific and sensitive SVs amongst many false positive calls from both technologies that can also serve as a high confidence benchmarking set.

## Materials and methods

### Genomic DNA samples

The MCF7 breast cancer cell line was obtained from American Type Culture Collection (ATCC), Manassas, VA. Cells were maintained in EMEM medium with 0.01 mg/ml of insulin and 10% fetal bovine serum (FBS). The cells were incubated at 37°C and in a 5% CO_2_ humidified environment.

The primary tumor tissue was purchased from BioIVT (https://www.bioivt.com/) and was available as a fresh frozen sample. The sample is a triple negative breast cancer primary tissue with 50% tumor content based on histopathological examination. The data was analysed anonymously.

### cWGS

DNA from MCF7 and the primary tumor sample was extracted with Qiagen’s DNeasy blood and tissue kit (Qiagen, Hilden, Germany). Whole genome libraries for NGS were prepared by fragmenting 1 μg genomic DNA to achieve an average fragment size of 550 bp. Subsequently, the library was prepared using KAPA hyper prep kit (Roche, Basel, Switzerland) using 8 bp single-index NEXTflex DNA barcodes and sufficient library yield was achieved by 4 cycles of PCR. Leftover adaptors were removed with 1X bead purification performed with Agencourt AMPure XP beads (Beckman Coulter, Brea, USA). The Qubit dsDNA HS assay kit (Invitrogen, Carlsbad, USA) and Bioanalyzer high sensitivity DNA kit (Agilent Technologies, Santa Clara, USA) were used for quality control. The libraries were sequenced on Illumina’s NovaSeq 6000 platform with S2 Reagent Kit for 300 cycles with a sequencing length of 2x150 bp (paired-end reads sequencing) with coverage as in S1 Table.

### 10XWGS

HMW genomic DNA was extracted from MCF7 and primary tumor tissue with MagAttract HMW DNA kit (Qiagen, Hilden, Germany). With 1 ng of HMW DNA, 10X Chromium reagents and gel beads library was prepared using the 10X Genomics Chromium genome reagent kit V2 user guide. Initial library construction takes place within droplets containing beads with unique barcodes. During library construction, a unique barcode (16 bp in length) is incorporated adjacent to Read-1. Final libraries were quantified on the Qubit using dsDNA HS assay kit (Invitrogen, Carlsbad, USA) and fragment length was determined using Bioanalyzer high sensitivity DNA kit (Agilent Technologies, Santa Clara, USA).

### Prediction of SVs from cWGS

The Illumina paired-end reads were aligned to the GRCh38 reference genome using BWA-MEM (version 0.7.17) [15], duplicates were removed using Samblaster v0.1.24–0 [16] and alignment files were sorted using Samtools v1.3.1 [17]. We referred to two review studies [18,19] for the selection of tools for prediction of SVs from cWGS. An ensemble of tools was chosen for better sensitivity and specificity that utilized multiple sources of evidence like discordant read-pairs, split reads, read depth and local *de novo* assembly. Since there is no single ensemble of tools that outperforms other ensembles [18], we selected three tools based on their popularity, easy usability, prediction of all SV types that can also be predicted by 10XWGS tools and inclusion of an assembly based tool. This ensemble included Delly (v0.7.6) [20], Lumpy (v0.2.13) [21] and SvABA (v0.2.1) [22]. All these tools utilize discordant read-pairs and split-reads, while Delly also utilizes read-depth and SvABA utilizes local *de novo* assembly. After the predictions from all the tools, SVs of the same type (deletion, duplication, inversion and translocation), sharing the same orientation (3’to5’, 5’to3’, 3’to3’ 5’to5’) and breakpoints within a 500-bp window were merged as a single SV call. This window size was selected as short-fragment sequence analysis can confidently relate breakpoints that are within the median fragment size (~500 bp) [23]. The CNVs predicted only by read-depth methodology were not analysed here, as exact breakpoints necessary for further comparison could not be inferred. In order to maximize sensitivity we considered all high quality calls (predicted with filter “PASS”) along with low quality calls (predicted without filter “PASS”) from all the three tools. Moreover, to assess the confidence level of calls from cWGS pipeline, we allotted high confidence calls to the predictions that were predicted with filter “PASS” by at least one of the tools.

### Prediction of SVs from 10XWGS

The sequenced linked-reads were analysed and processed using Long Ranger v2.2.2 wgs command with–somatic flag. The reads were aligned to the GRCh38 reference genome using Lariat and SNPs were predicted by freebayes v0.9.21-7-g7dd41db-dirty. The Long Ranger from 10X Genomics performs haplotype phasing and predicts SV after estimating a probability of barcode overlap between linked-reads and split reads for refining the breakpoints of rearrangements. The Long Ranger reports following types of SVs: deletion, duplication, inversion, translocation and some unresolved variants labelled as ‘Unknown-UNK’. The CNVs predicted only by read-depth were not considered for analysis here. For a fair comparison with cWGS pipeline and to maximize sensitivity, we included two more tools utilizing linked-reads for prediction of SVs. The tool NAIBR v1.0 also performs haplotype phasing and constructs a probabilistic model to find novel adjacencies using discordant read-pairs and split barcoded molecules from linked-reads sequencing [24]. While GROC-SV v0.2.5 [25] utilizes a similar approach as Long Ranger additionally with local assembly at breakpoints using linked-reads. All the high quality calls (reported with filtered “PASS”) and low quality calls (reported without filter “PASS”) were considered for the comparison. The SVs from three tools were merged with the same scheme followed for intersection by cWGS pipeline. In order to estimate the confidence level of SVs from 10XWGS pipeline, each call was allotted high confidence when predicted with filter “PASS” by at least one of the tools.

### Requantification of supporting reads for SVs

In order to evaluate the two technologies, we used an approach that quantifies the number of supporting reads for the SVs. The workflow (S1A Fig) involves construction of a synthetic genomic template from the sequence of reference genome. For SVs larger than 1 kb, a 1 kb template is constructed by retrieving 500 bp reference genome sequences to either side of the breakpoints, which are then fused according to the orientation of reported SV (S2 Fig). For SVs below 1 kb, the size of genomic template is reduced to atleast twice the size of SV. Next, short-reads are aligned to this synthetic genomic template with BWA-aln (version 0.7.17). From each SV alignment, we calculate the number of reads overlapping the fusion breakpoint for at least 15 bp (junction reads, JR) and read-pairs that span breakpoints (spanning pairs, SP). Only the reads with at least 70% of its bases aligning to the genomic template were considered for JR and SP. JR and SP were normalized as:

$$Normalized\;junction\;reads\;(JR)=\frac{Number\;of\;junction\;reads\;supporting\;SV}{Total\;number\;of\;reads}*10^{8}$$ (1)

$$Normalized\;spanning\;pairs\;(SP)=\frac{Number\;of\;spanning\;pairs\;supporting\;SV}{Total\;number\;of\;read-pairs}*10^{8}$$ (2)

Jointrequantificationsupport(JRS)=JR+SP (3)

The requantification support was calculated from reads from both the technologies. Since, cWGS samples were sequenced at higher coverage than 10XWGS samples, we downsampled cWGS reads for calculation of requantification support. Moreover, read-1 from 10XWGS contains a 16 bp barcode sequence. Thus, for calculation of requantification support we trimmed the reads to a length of 125 bp, thereby removing the barcode. JR, SP and JRS were labelled with their sources as cWGS or 10XWGS.

### GEM quantification for SVs

We also calculated the number of unique barcodes or GEMs containing read-pairs that support SVs reported from both the technologies. For this we used 10XWGS generated alignment file to first separate read-pairs that are aligned without a normal alignment FLAG. This was done using tool Samblaster v0.1.24–0 [16]. Next we counted number of unique barcodes or GEM (with BX tag in BAM file) that support a particular type and orientation of SV (S1B Fig). The unique GEMs were retrieved in the window w_i_ around breakpoints. The window size was selected as the ratio of average molecule length and N50 linked-reads per molecule from 10XWGS experiment. The GEM count was normalized as:

$$Normalized\;GEM\;count=\frac{Number\;of\;GEM\;supporting\;SV}{Total\;GEM\;detected}*10^{6}$$ (4)

### Annotation of SVs and comparison from cWGS and 10XWGS

Each breakpoint of the SV was annotated with repeat region masked in RepeatMasker and poor mappability region [26]. In order to investigate the advantage of 10XWGS technology, we also calculated local coverage around the breakpoints in a window of size 400 bp for each SV. This was calculated using samtools pileup command and the local coverage was normalized by average coverage of the sequenced sample.

The SVs with size greater than 50 bp from both technologies were compared based on their breakpoint positions (within a window of 500 bp), type and orientation. As the 10XWGS pipeline reports inversions and duplications with size greater than 10 kb only, comparison was performed for those size ranges of inversions and duplications.

### PCR confirmation of SVs

Some of the SVs that were common and uncommon between the technologies were selected for validation by PCR. We randomly selected a comparable number of candidate SVs from shared, 10XWGS only and cWGS only identified SVs. PCR primers were designed according to the predicted breakpoint spanning the junction site of the rearrangement with one primer positioned upstream and the corresponding primer downstream of the fusion. The genomic template for primer designing was produced according to the type and orientation of SV (S3 Fig).

Each PCR contained 10 ng sample DNA and primers with a final concentration of 0.333 μM each. The final volume was 30 μl using HotStarTaq Master Mix Kit (QIAGEN Cat.No. 203443) and 3 step-PCR with an annealing temperature of 60°C for 40 cycles according to the manufacturer’s recommendation.

Subsequently, the PCR products were analyzed on a QIAxcel capillary gel electrophoresis instrument using QIAxcel DNA Screening Kit (QIAGEN Cat. No. 929004). For alignment and size determination, a 15 bp / 500 bp marker (QIAGEN Cat.No. 929520) was used.

### Sanger sequencing

To further confirm the PCR products, Sanger sequencing was performed in forward and reverse direction with primers used for the PCR. Samples were sent to Eurofins genomics (https://www.eurofinsgenomics.eu/) for sequencing.

### Statistical analysis

All statistical tests were performed in R (version 3.6). The nonparametric Wilcoxon Rank sum test was used to compare positive and negative groups of PCR validated SVs. It was also used to compare local coverage around the breakpoints of SV derived from cWGS and 10XWGS alignments. While pairwise Kruskal-Wallis test was used to compare three groups of SVs: common SVs (predicted by both the technologies), only 10XWGS SVs (predicted only by 10XWGS) and only cWGS SVs (predicted only by cWGS).

### Logistic regression model

Two logistic regression models were trained for filtering true positive calls from the cWGS and 10XWGS technology respectively. The features common between models were type of SVs (deletion, duplication, inversion and translocation), normalized junction reads (JR), spanning read-pairs (SP), size of the SV and local coverage around the positions. These were calculated from reads originating from the respective sequencing technology. Comparatively, the 10XWGS model also included GEM count as another feature. Only the SVs internally tested by PCR and predicted with respective technology were used for training and testing the model (for cWGS: Positive SVs = 178, Negative SVs = 75; and for 10XWGS: Positive SVs = 131, Negative SVs = 106). The respective data set was divided in 70:30 ratio as training and test data set. The performance of models was measured on test data chosen with bootstrap resampling with 10 resamples (S17 Fig). Since the training data set for cWGS model was unbalanced, we also tested the performance of models with different type of sampling strategies (down sampling, up sampling and SMOTE). However, different samplings to balance the unbalanced data did not improve the performance of original cWGS model. Hence, we trained the cWGS model with unbalanced data only. Finally, we predicted true SVs as the ones predicted by either model with probability greater than 60%. The training of the classification model was carried out with the package caret in R v3.6 and importance of individual features was calculated with varImp function of caret. The varImp function calculates importance based on the absolute value of their t-statistics. The relative importance of features was calculated using dominance analysis [27] that derives importance of one feature over others by creating a subset of models with different combinations of features.

## Results

### cWGS and 10XWGS predict different numbers and classes of SVs

We compared cWGS and 10XWGS in terms of the numbers and classes of SVs predicted in two samples: a breast cancer cell line (MCF7) and a primary breast cancer sample. MCF7 and primary breast cancer sample was sequenced with 51X and 92X by cWGS technology. Their sequencing coverage was 17.4X and 17.7X respectively, by 10XWGS technology. The physical fragment coverage achieved by 10XWGS technology was 87X and 88.5X for MCF7 and primary breast cancer (nearly equivalent to average coverage of samples sequenced by cWGS) (S1 Table).

SVs were predicted by combining calls from an ensemble of three SV detection tools for cWGS data (SvABA, Delly and Lumpy) and three tools for 10XWGS data (Long Ranger, NAIBR, GROC-SV). The set of cWGS tools included Delly and Lumpy that use discordant read-pairs, split reads for detection of SVs and are widely accepted tools. Additionally, SvABA, a local assembly tool, was also included as Cameron *et*. *al*. [18] proposed an ensemble with a local de novo assembly tool for best performing collection of cWGS tools for SVs. Considering this, we created an ensemble of 10XWGS tools that use discordant read-pairs, split barcode molecules, barcode overlap and local de novo assembly. This included Long Ranger, GROC-SV and NAIBR. All the high and low quality SV calls from tools were considered and merged according to the type, orientation and their breakpoints. They are also referred to as high and low confidence calls respectively.

**Fig 1:**
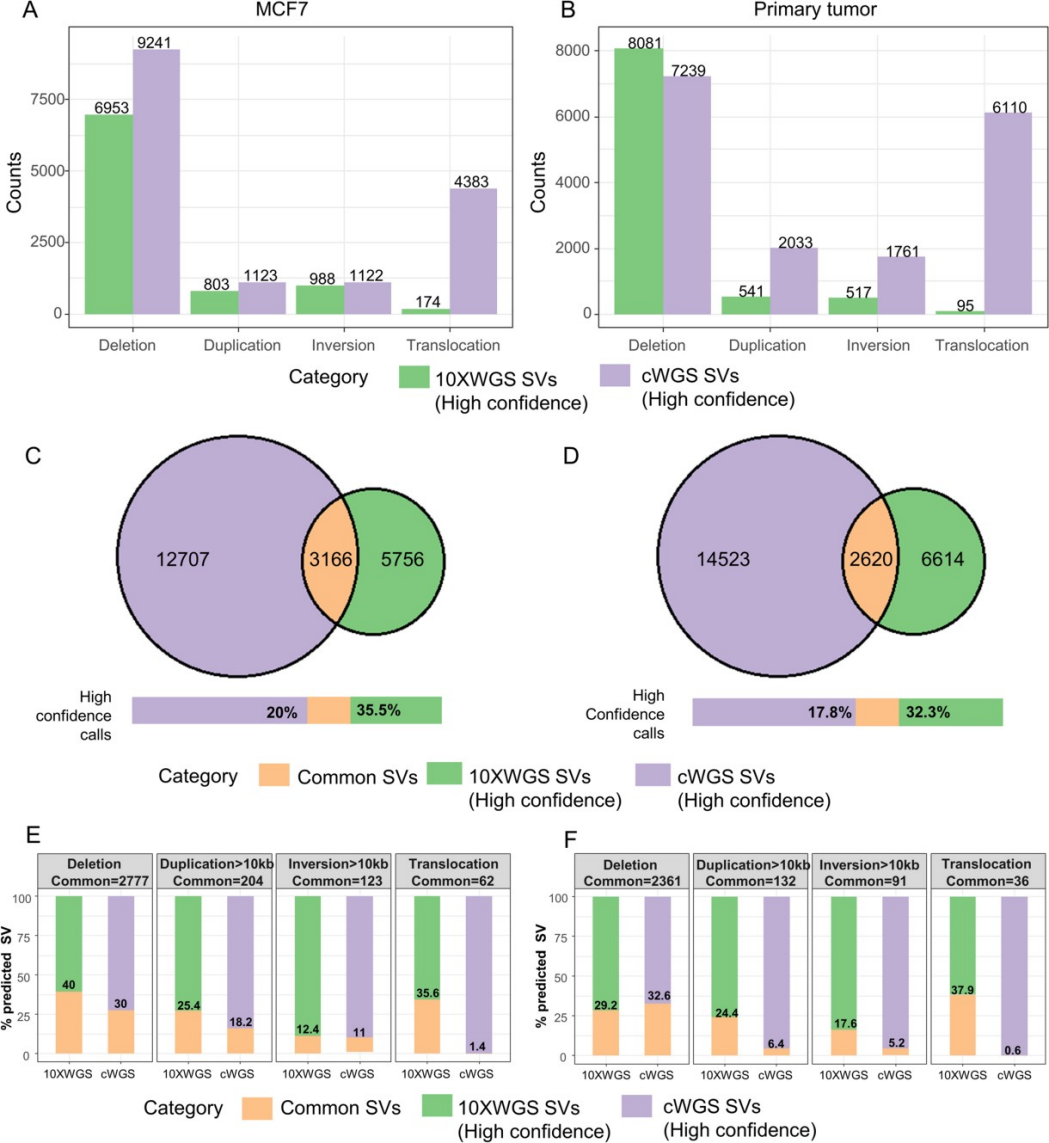
cWGS and 10XWGS predict a variable number of SVs with low proportion of common predictions. (A and B) Number of different types of SVs predicted with high confidence by cWGS and 10XWGS pipelines for (A) MCF7 and (B) primary breast tumor. (C and D) Number of high confidence SVs commonly predicted by both technologies for (C) MCF7 and (D) primary breast tumor. (E and F) Percentages of the indicated high confidence SVs commonly predicted by the two approaches for (E) MCF7 and (F) primary breast tumor.

First, we investigated the different types of SVs identified by the cWGS and 10XWGS pipelines in both samples (Figs 1 and S4). There was significant difference in the number of different types of SVs predicted by the two pipelines (irrespective of high or low confidence calls). The ensemble of cWGS tools predicted comparatively higher number of all SV types (especially translocations). When looking in more detail into different size ranges, both the cWGS and 10XWGS pipelines identified deletion of all size range (S4E and S4F Fig) but the 10XWGS pipeline predicted nearly 5 times less deletions. The highest number of deletions in the cWGS pipeline came from low quality calls of SvABA while in the 10XWGS pipeline they came from high quality calls of Long Ranger (S4E and S4F Fig). Moreover, the 10XWGS pipeline predicted about 6 times less duplications in comparison to the cWGS pipeline when we consider both high and low confidence calls. This can also be attributed to the fact that tools in the 10XWGS pipeline predicted duplications with size>10 kb only (S4E and S4F Fig). However, tools in the cWGS pipeline predicted all sizes of duplications where most of them are low quality calls from SvABA and Delly (S5B and S6B Figs). Similar to the duplications, the 10XWGS pipeline predicted inversions greater than 10 kb only. However, ~99% of inversions in the 10XWGS pipeline are predicted as low quality calls from Long Ranger that lie in the size range of 10–100 kb. This seems to be an attribute of Long Ranger methodology as other tools (NAIBR and GROC-SV) did not predicted such high number of inversion (S4, S5C and S6C Figs). The 10XWGS pipeline detected 100–200 fold fewer SVs with size >100 kb compared to the cWGS pipeline (S4E and S4F Fig). Since the 10XWGS pipeline generates long-range information from short-reads, it should be able to minimize alignment-based artefacts and therefore have a specificity advantage especially for those larger events.

The most remarkable difference in numbers was observed for translocations (Figs 1A, 1B, S4A and S4B). The cWGS pipeline predicted a much higher number of translocation in comparison to the 10XWGS pipeline. Majority of these translocations in the cWGS pipeline are contributed by low quality calls from SvABA and Delly (S5D and S6D Figs), which can be result of imprecise breakpoints, low mapping quality of reads, lower support in terms of discordant read-pars or split read etc. Moreover, as for other large SVs >100 kb from the 10XWGS pipeline, long-range information and low false discovery rate (FDR) translated into more precise number of translocations. Overall, the order of magnitude of predicted SVs is comparable between the cell line and the primary tumor sample, but the overlap is low.

### Debarcoded and downsampled MCF7 SVs

Since the average genomic coverage of cWGS MCF7 sample was higher than 10XWGS MCF7, we tested SV prediction pipeline on downsampled cWGS reads (downsampled MCF7, equivalent genomic coverage as 10XWGS). We also tested a strategy to use cWGS tools with 10XWGS linked-reads. For this, barcodes in 10XWGS linked-reads were trimmed and the reads were processed in cWGS pipeline (debarcoded 10XWGS MCF7). It was observed in S7 Fig, the overall number of predicted SVs is reduced in the downsampled and debarcoded samples. This was especially true for the only cWGS predicted SVs (drops to ~50% and 70% respectively), while the number of common remained stable (~99.1% for debarcoded and 85.3% for downsampled samples). It is also evident from the debarcoded sample that allows analysis of exactly the same reads without linkage information in cWGS pipeline. However, the cWGS pipeline with debarcoded reads predicted very high number of small size SVs (size <1 kb, as seen in S7A Fig). This can be a ripple effect of reads from a different technology processed by algorithms designed for alternate technology. For further analysis, we decided to stick with the sequenced cWGS data sets whose genomic coverage matches physical coverage of the 10XWGS data.

### A small fraction of predicted SVs is common to both cWGS and 10XWGS pipelines

We compared the calls between both technologies according to the breakpoints (within a window of ±500 bp), type and orientation of SVs: Fig 1C and 1D depicted the rather small overlap between both technologies for high confidence calls. This overlap was even smaller when low confidence calls were also considered in S4C and S4D Fig. Since we pool SV calls from multiple tools in both cWGS and 10XWGS pipelines, it is expected to have a high number of false positive predictions but higher true positive as well. However, this aggregation of the cWGS calls should result in high sensitivity and have rather higher overlap with 10XWGS calls. Contrastingly, the majority of high confidence 10XWGS calls do not overlap and only 35.5% and 32.3% of 10XWGS-predicted SVs were also predicted by the cWGS pipeline for MCF7 and the primary tumor, respectively. This raises the question of whether 10XWGS predicts SVs inaccessible by cWGS technology or whether the 10XWGS suffers from a high FDR. Or, vice versa, cWGS technology is more sensitive than 10XWGS, which misses many SVs.

There were differences with respect to different types of SVs (Fig 1E and 1F). Nearly 35.6% and 37.9% of high confidence translocations as predicted by 10XWGS were also predicted by cWGS from MCF7 and primary tumor respectively. The overlap increased slightly to 48.2% and 53.2% for MCF7 and primary tumor respectively, when low confidence calls were also considered (S4G and S4H Fig). Conversely, the percentage of common translocations by cWGS was extremely small (1.4% for MCF7 and 0.6% for primary tumor) due to the much higher number of predicted events. This implies that the cWGS pipeline is possibly sensitive, but has a very high FDR especially for translocations.

Additionally, we investigated whether high confidence calls by either pipeline are enriched among the common SVs. As depicted in S8A Fig, 41.1% and 35.1% of high confidence 10XWGS calls in MCF7 and primary tumor, respectively, were common between both the technologies. And, only 1.6% and 1.3% of low confidence 10XWGS calls were common in MCF7 and primary tumor, respectively. Comparatively, 20.4% and 15.5% of high confidence cWGS calls in MCF7 and primary tumor, respectively, were common between both the technologies. But, only 0.18% and 0.11% of low confidence cWGS calls were common in MCF7 and primary tumor, respectively. This indicates that common calls are high confidence calls from respective technologies. Moreover, 38.4% and 54.9% of calls predicted by all three tools in the cWGS dataset for MCF7 and the primary tumor (S8C and S8D Fig) were also predicted by 10XWGS. Comparably, all the calls predicted by all three tools in 10XWGS were predicted by cWGS pipeline. However, as depicted in S5, S6 and S8E Figs, very few calls were commonly predicted by all three tools in the 10XWGS pipeline. This is exemplified by the fact that 50% of common calls were predicted by all three tools in cWGS pipeline, while only 1.2% of common calls were predicted by all tools of the 10XWGS pipeline for MCF7.

### Common SVs have higher read and GEM coverage

**Fig 2:**
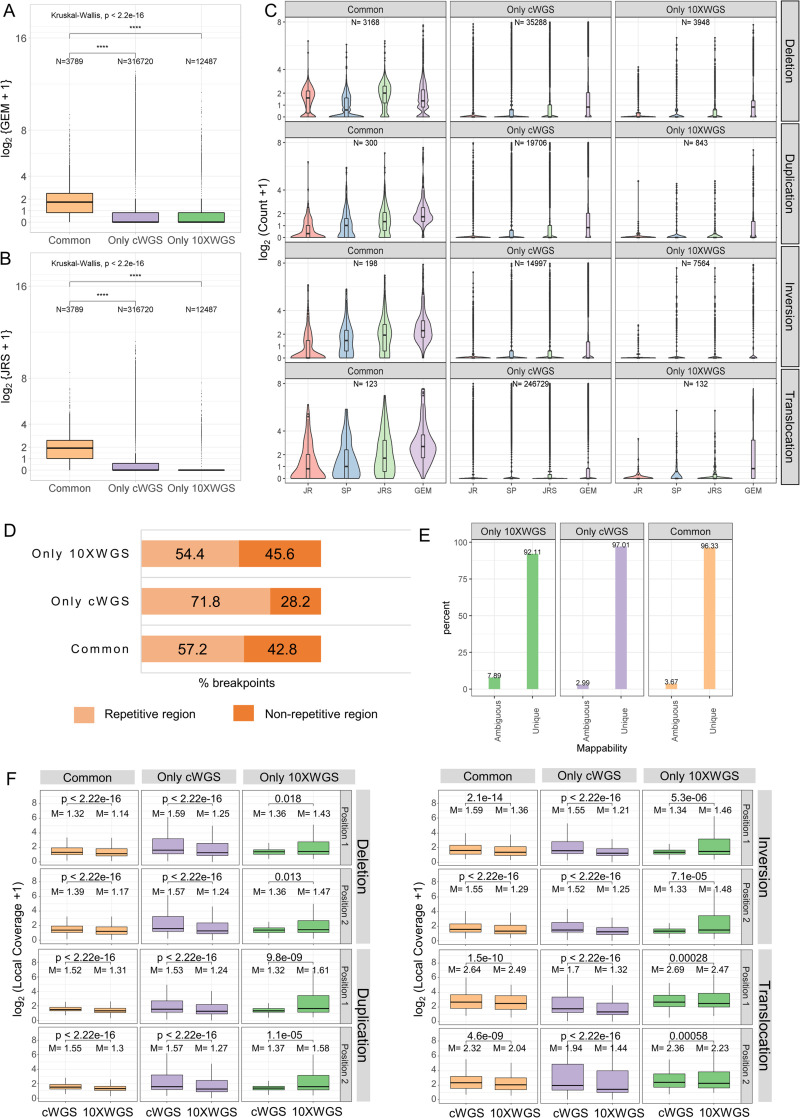
Requantification support and GEM coverage for SVs common between cWGS and 10XWGS is higher than that predicted by a single technology. (A) Distribution of GEMs containing SVs that were predicted by both the technologies (common) or only by one technology (only cWGS or only 10XWGS) for MCF7. (B) Shown is the combined requantification support (JRS) as the sum of junction and spanning reads from cWGS data for common SVs and SVs predicted only by cWGS or 10XWGS for MCF7. p-values were calculated using Kruskal-wallis test and pairwise Wilcoxon rank sum test. **** represents a p-value <0.0001. (C) Comparison of requantification support (Junction reads-JR, Spanning pairs-SP, JRS = JR+SP) and GEMs for different type of SVs that are common between technologies and only predicted by 10XWGS or cWGS for MCF7. The black lines in the boxes represent median (centre line), upper quartile (upper line) and lower quartile (lower line), respectively. The area of violin plots is scaled to the number of observations. (D) Percentage of breakpoints of high confidence SVs from two technologies covered by repetitive regions. (E) Percentage of breakpoints of high confidence SVs from two technologies covered by unique mappability regions. (F) Distribution of normalized local coverage around the positions of high confidence SVs (size >10 kb), calculated from cWGS and 10XWGS aligned reads respectively. p-values were calculated by pairwise Wilcoxon rank sum test and ‘M’ is median of normalized local coverage.

Since junction reads (JR), spanning pairs (SP) from both the technologies and unique barcodes (GEM) from linked-reads sequencing are the main cues for true SVs, we quantified them by a common computational approach for all identified SVs (Eqs 1, 2, 3 and 4). This allowed us to investigate differences in different categories of SVs: calls predicted by both the technologies (common SVs), calls predicted only by cWGS technology (only cWGS SVs) and calls predicted only by 10XWGS technology (only 10XWGS SVs). Common SVs had a significantly higher median count for JRS (median = 1.9) and GEM (median = 1.73) in comparison to only cWGS SVs (JRS: median = 0, GEM: median = 0) and only 10XWGS SVs (JRS: median = 0, GEM: median = 0) (Fig 2A and 2B). This inference was also drawn when different types of SVs were considered separately (Figs 2C, S9 and S10). Furthermore, since there might be differences in the libraries of the two technologies, we also calculated requantification support using 10XWGS reads. As depicted in S9–S12 Figs, we can draw same inference irrespective of the source of reads (cWGS or 10XWGS). Conclusively, regardless of the used technology and the used metric (JRS or GEM), common SVs were in all situations better supported.

Overall 63.5% of common SVs were supported by at least two JRS from cWGS data for MCF7. While 9.9% of only cWGS SVs and 6.6% of only 10XWGS SVs had at least a JRS of two from the respective technology. When high confidence calls were considered from the respective pipelines, 31.7% of only cWGS SVs and 14.6% of only 10XWGS SVs had at least a JRS support of two from their respective technology. It is surprising to note that the only cWGS SVs also had support from 10XWGS linked-reads: 30.4% of only cWGS high confidence calls were also supported with at least a JRS of two calculated from 10XWGS linked-reads. Comparatively, only 10.8% of only 10XWGS high confidence calls had at least a JRS of two from cWGS data. It is somehow expected that each technology gives overall higher support to the SVs identified by them. However, we observed that a higher fraction of high confidence SVs only predicted by cWGS still had higher requantification support in comparison to the ones predicted only by 10XWGS. This implies that many of the SVs predicted only by the cWGS pipeline do have evidence in the 10XWGS sequenced data (overlapping GEMs, JRs and SPs) but the 10XWGS tools did not identify them (Figs 2A, 2B and S11). Vice versa, high confidence SVs predicted only by 10XWGS have overall lower support from both the technologies. The same observations that are described here for MCF7 were also made for the primary tumor sample (S10 and S12 Figs). This data indicated that common events are most likely enriched for true positive events. Nevertheless, additional true positive events are contained in only cWGS SVs while only 10XWGS SVs contributes a lower number of true SVs.

To further characterize differences between both sequencing technologies, we annotated each breakpoint of the SVs for repetitive regions and ambiguous mappability regions. It is well established that short-reads originating from repetitive regions are often misaligned [3]. Considering the breakpoints of high confidence SVs from both pipelines in Fig 2D, it was observed that breakpoints of 57.2% common SVs and 54.3% only 10XWGS SVs are inside a repetitive region with majority being in SINE and LINE (S13B Fig). However, for only cWGS SVs, 71.8% of the breakpoints were inside repeats where satellite and simple repeats contributed towards 49% of the breakpoints. This indicates that a high fraction of these calls may be false positive calls due to misalignment. Secondly, when considered all the SVs (both high and low confidence ones), the percentage of breakpoints in ambiguous mappability regions were higher for only cWGS SVs than only 10XWGS SVs (S13C Fig). When only high confidence calls were considered in Fig 2E, more than 90% of breakpoints were in unique mappability regions. Overall, cWGS and 10XWGS technology contributed fewer SVs with breakpoints in low complexity and LTR regions, while SVs with breakpoints in SINE and LINE elements were common in both.

The 10XWGS technology links short-reads to their larger size DNA fragment and is assumed to improve local physical coverage of SV breakpoints. Thus, we compared the normalized local coverage derived from both cWGS and 10XWGS aligned reads for all SVs greater than 10kb. When we considered all SVs (both high and low confidence calls), we did not observe a significant difference in local coverage for 10XWGS only calls between the two technologies (except in inversions) (S13D Fig). However, in Fig 2F we considered only the high confidence calls and had shown that the local coverage in only 10XWGS SVs is higher when 10XWGS aligned reads were considered (except in translocations). Moreover, common and only cWGS calls had higher local coverage from cWGS aligned reads. This indicates that prediction of additional SVs from 10XWGS might indeed be the result of improved coverage, with these SVs missed by cWGS sequencing.

### PCR confirms high specificity of common SVs

**Fig 3:**
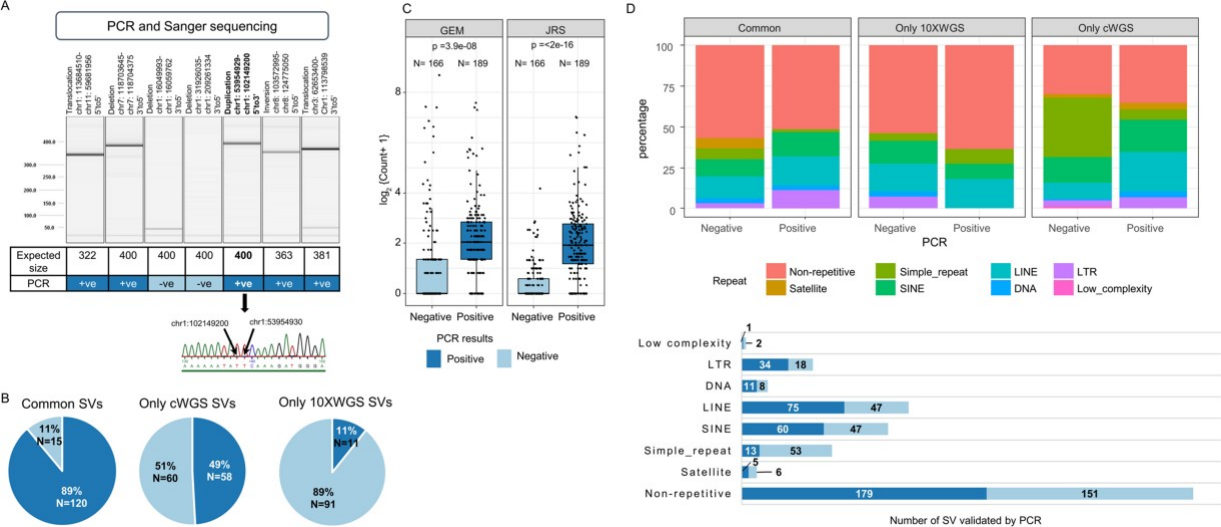
Orthogonal validation of SVs using PCR and Sanger sequencing. (A) SVs within the MCF7 dataset were selected for validation by PCR and Sanger sequencing. From the PCR-amplified products, a subset was further confirmed by Sanger sequencing. Shown are representative results involving seven SVs. (B) Number and percentage of PCR-validated SVs for the three categories: SVs common between cWGS and 10XWGS (common SVs), SVs only predicted by cWGS pipeline (only cWGS SVs) and SVs only predicted by 10XWGS pipeline (only 10XWGS SVs) are shown. (C) The difference in normalized counts of combined requantification support (JRS from cWGS reads) and GEM for PCR-validated SVs is shown. Each data point represents counts for PCR tested SVs and box-and-whisker plots represent lower quartile, median and upper quartile. p-values were derived from Wilcoxon rank sum test. (D) Percentage and number of repetitive element classes in PCR validated SVs for three categories: common, only cWGS and only 10XWGS SVs.

We validated a comparable number of randomly selected common and uncommon SVs from the three categories: 135 common SVs, 118 only cWGS SVs and 102 only 10XWGS SVs (S2 Table). The orthogonal validation was performed with PCR and Sanger sequencing of SVs from MCF7. Fig 3A exemplifies the PCR validation results for seven SVs: Five SVs led to amplification of a product of expected size and were therefore determined as positive. Additionally we selected a subset of positive amplicons for Sanger sequencing for confirmation of the sequence across the breakpoint, as depicted in Fig 3A. In total, we confirmed 36 out of 42 amplicons by Sanger sequencing. The remaining six amplicons had poor quality sequence traces and could not be analysed.

The pie charts in Fig 3B illustrated the confirmation rate for SVs from the respective categories. 89% of common SVs were confirmed by PCR. This indicated that the combined approach of 10XWGS and cWGS is highly specific for the prediction of SVs. Only 15 common SVs were not confirmed by PCR. We followed these up in detail by manual inspection of the sequence alignment from cWGS data. Here, we observed that either the breakpoints were in repetitive regions, SVs lacked proper read support, reference genome region was not annotated or the SV events shared the same breakpoint i.e. they were complex in nature (S14 Fig). In contrast, the confirmation rate for SVs only predicted by cWGS and 10XWGS dropped to 49% and 11% respectively. This confirms that the 10XWGS pipeline is prone to prediction of false positive SVs. We further investigated the PCR validation rate for SVs that are an overlap between tools from respective pipelines. S15 Fig shows that cWGS SVs predicted by the consensus of all tools have a maximum PCR confirmation rate (i.e. 84.4%). This is in agreement with the popular approach of considering consensus SV calls from multiple tools to reduce false positive calls by cWGS technology. Similarly, consensus predictions from the 10XWGS pipeline had 84% confirmation rate. The confirmation rate for consensus deletions and duplications by 10XWGS was 100% and 60% respectively. However, confirmation rate for duplications predicted by two tools of the 10XWGS pipeline was higher at 84.2%. A similar trend of most confirmation rates for calls predicted by all three tools of the 10XWGS pipeline was followed for inversions (75%) and translocations (100%) and also by all the SV types in cWGS pipeline.

In order to confirm that requantification support and GEM counts can serve as a metric to filter out true positive SVs, we plotted their counts for PCR-tested SVs in Fig 3C. The PCR-positive SVs had significantly higher requantification support (JRS) and GEM coverage in comparison to ones that are tested PCR-negative. This was also true for requantification support calculated using 10XWGS reads (S16 Fig). Moreover, we compared the confirmation rate for PCR validated SVs with respect to the repeat class of breakpoints in Fig 3D. It was observed that validation rate for SVs in simple repeats was lower, while differences in validation rates for other classes could not be derived. Moreover, a higher percentage of SVs only predicted by cWGS in simple repeats could not be confirmed by PCR. As expected, this indicates that cWGS pipeline cannot resolve SVs in simple repeats.

**Fig 4:**
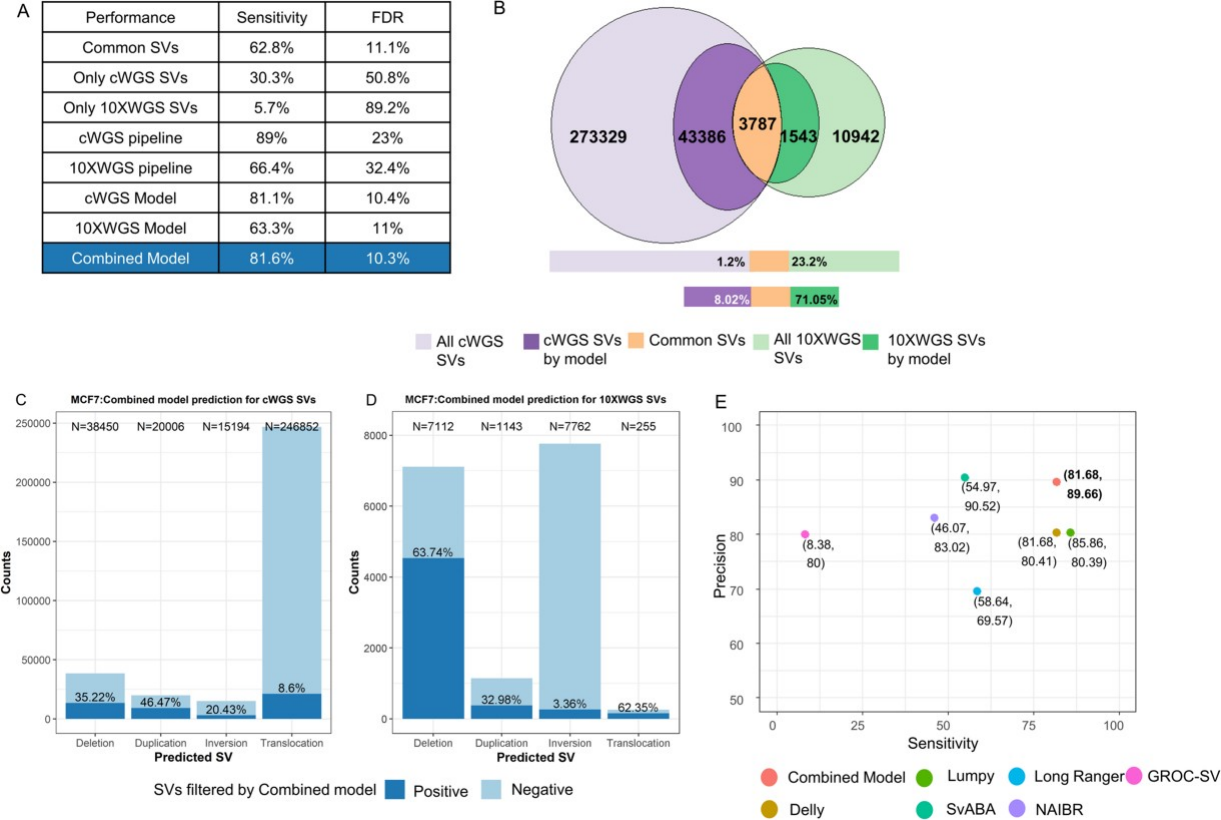
Prediction of SVs by trained models for the cWGS and 10XWGS technology. Two logistic regression models were trained on PCR tested SVs from the respective technologies. (A) The table depicts the performance of different categories of SVs or technologies derived from PCR tested SVs. (B) Numbers and percentage of SVs common between the technologies before (lighter shades) and after (darker shades) applying the respective trained models. (C) Number of SVs predicted by the cWGS technology within the MCF7, and percentage predicted positive by the combined models. (D) Number of SVs predicted by the 10XWGS technology within the MCF7, and percentage predicted positive by the combined models. (E) Plot for performance of combined model and all other tools on internally validated SVs.

For a direct comparison of these two technologies, we calculated the sensitivity and FDR using PCR-tested SVs in Fig 4A. The SVs predicted by both technologies had 62.8% sensitivity with a very low FDR of 11.1%. However, SVs only predicted by one of the technologies had much higher FDR. Overall the cWGS pipeline had high sensitivity (89%) but with a high FDR of 23%. Comparatively, the 10XWGS pipeline had lower sensitivity (66.4%) with an even higher FDR of 32.4%. This indicated that even the 10XWGS pipeline is prone to high FDR and requires more stringent filtering criteria to further enrich true positive SVs.

### Enrichment of true positive SV calls using requantification support and GEM count

The data indicates that for a highly specific and sensitive prediction of SVs a combined approach using cWGS and 10XWGS prediction data might be advisable. Nonetheless, we created prediction models for both the technologies independently to improve the prediction as much as possible for situations when only data from one of the technologies is available. Additionally, we combined all predictions into a unified approach to offer best sensitivity and FDR when both analyses are available. Initially, we also tested a simple filtering approach based on the number of supporting reads to enrich for true positive events, but observed poor sensitivity as there is no clear separation between PCR positive and negative SVs (Fig 3C).

To this end, we generated two logistic regression models using PCR validated data, one for the 10XWGS data and a second one for the cWGS data. In Fig 4A, we measured sensitivity and FDR of both the models based on PCR tested SVs. It was evident that FDR reduces drastically after applying the trained models. Predictions from the cWGS model and the 10XWGS model show a reduced FDR from 23% to 10.4% and from 32.4% to 11%, respectively. However, this came at the cost of reduced sensitivity, which decreased from 89% to 81.1% for the cWGS model and from 66.4% to 63.3% for the 10XWGS model. Moreover, SVs predicted by both technologies had a very low FDR but with sensitivity lower than for the overall cWGS pipeline (as shown by PCR). Application of both models increased the percentage of SVs common between both the technologies from 1.2% to 8.02% for cWGS and 23.2% to 71.05% for 10XWGS in MCF7 (Fig 4B). This is another evidence for the decrease in FDR achieved by both the models. A similar increase in overlap was also seen in an independent primary tumor sample (S18 Fig).

All three approaches (common SVs, cWGS model and 10XWGS model) aim to enrich different subset of true positive SVs. We therefore considered all these calls in a combined model for best sensitivity and low FDR and tested its performance on PCR validated SVs. To this end, we observed a reduced FDR to 10.3% and a high sensitivity of 81.6% similar to the cWGS model (Fig 4A). Application of the integrated approach made a dramatic difference on the overall landscape of predicted SVs from cWGS and 10XWGS (Figs 4C, 4D and S18): The combined model filtered out 85.3% and 86.9% of total calls in MCF7 and primary tumor respectively. Moreover, the most significant reduction in MCF7 was observed for translocations from cWGS where we observed a reduction to 8.6% of total calls. In case of the 10XWGS technology, we observed a maximum reduction of inversions to 3.36%.

Overall, the combined model gathered good sensitivity and precision for overall performance against the other tools (Fig 4E), for internal PCR validated SVs. The combined model achieved 81.68% sensitivity and 89.66% precision on the full MCF7 sample. Comparatively, only Delly and Lumpy had comparable sensitivity of 81.68% and 85.85% respectively. However, their precision was around 9% lower than for the combined model. SvABA had shown slightly superior precision with 90.52%, but at the cost of much lower sensitivity (54.97%). Therefore, the combined model offered best overall performance tradeoff in terms of sensitivity and precision. Compared to the 10XWGS tools the advantage was even more apparent. The combined model also greatly reduced cWGS only calls predicted in simple repeat and satellite regions (compare S19 to S13 Fig). Therefore, 10XWGS only calls contained a higher fraction of SVs in simple repeat regions. This is in-line with the notion that 10XWGS offers superior performance in these low complexity regions due to use of long range information. Of note, even when only cWGS or 10XWGS data is used, our established models can still compete well with the other tools of the respective technology. Moreover, we also compared the performance of the combined model against other tools, when the reads were downsampled or debarcoded. As depicted in S20 Fig, results on downsampled and debarcoded datasets had shown decreased sensitivity for all tools, but are otherwise very comparable.

### Benchmarking combined model

We tested the performance of combined logistic regression model also on previously validated SVs in MCF7. A list was gathered from Li *et*. *al* [28] (external study 1) that included 183 SVs of size greater than 500 kb. These calls were detected by the tool Weaver and confirmed with optical mapping. Another set of 70 validated SVs was collected from Hillmer *et*. *al* [29] (external study 2) that was detected by a long-span paired-end-tag sequencing approach and was validated by PCR. We also benchmarked the model with germline SV calls as available in gnomAD study to confirm shared germline events present in MCF7 [30].

On the external study 1 data set, the combined model achieved sensitivity of 76.69% which was lower than Delly (94.54%) and Lumpy (95.06%) (S20 Fig). However, it was superior in terms of sensitivity to SvABA (72.13%), Long Ranger (26.23%), NAIBR (34.97%) and GROC-SV (10.93%). Here, the results differ from our own data, but this study only contains large structural variants and therefore offers insights into this subset of SVs only. For the external study 2 calls, the combined model achieved a sensitivity of 84.29%, which is comparable to Delly (85.71%) and Lumpy (84.29%). However, it was superior in terms of sensitivity to SvABA (58.57%), Long Ranger (62.86%), NAIBR (70%) and GROC-SV (11.43%). For this data set we observed similar sensitivities to our data set. When considering the germline SVs from gnomAD study as another set of validation, the calculated sensitivity was very small as only a small subset of known germ line SVs is expected in in MCF7 cell line (S20A, S20B and S20C Fig). Nevertheless, the combined model achieved better sensitivity in comparison to all other tools. When considering all gnomAD germline SVs present in MCF7, the combined model maintains good sensitivity compared to all unfiltered predictions (2629/3076 ~ 85.47%; S20F Fig). When we look at SV predictions with downsampled and debarcoded reads, then the combined model consistently performed better than all the tools (S20B, S20C, S20D and S20E Fig). This shows the robustness of the combined model for even lower genomic coverage samples. When calculating precision based on these external datasets, we observed artificially poor values for our combined model (S3 Table). However, these datasets only partially reflect the entire range of SVs (e.g. limited size range, only germ line SVs). Therefore, any general approach towards SV prediction will perform poor in such an analysis.

Taken together, the here presented logistic regression model provides a sensitive and accurate filter to predict true positive SVs. The model can also be utilized for reads from only one technology (cWGS or 10XWGS), but of course, at the cost of reduced sensitivity.

## Discussion

Structural variations can have diverse functional impacts in humans; therefore, when performing genomic analysis of any disease state, it is imperative to find true positive SVs that might be associated with a certain phenotype. A popular approach to identify SVs is the cWGS technology, which suffers from high FDR (up to 85%) and varying sensitivity (30–70%) [31–33]. Here, we aimed to boost sensitivity for SV detection by integration of multiple bioinformatics tools, which is a common practise utilized in many studies [19,33,34]. Typically this comes at the cost of high FDRs. In order to reduce the FDR, many studies consider only the consensus from multiple bioinformatics tools [19,33,34]. In our analysis, we could show that the focus on SVs that are found by multiple tools can indeed achieve low FDR, but at the cost of much reduced sensitivity. This shows that better approaches are needed to enrich true positive SVs in such scenarios.

More recently, the development of 10XWGS technology seem to offer an elegant solution by taking into account long-range mapping information for the prediction of SVs. Our validation data had shown a relatively high FDR of 10XWGS for SVs which is improved when only high confidence calls are considered. However, compared to cWGS sequencing, 10XWGS had lower sensitivity when considering all types of SVs. This is in line with previous studies that reported varying sensitivity of 35–88.4% and moderate FDR of 50% for the 10XWGS technology [10,35]. Since 10XWGS is the latest technology, there are currently fewer algorithms available for the analysis of data. Nevertheless, we compared the performance of set of those algorithms against cWGS tools here. Contrary to previous studies, where performance metrics were derived from publically available datasets that are limited in type and size of SVs and are derived from diploid genomes, we presented a comprehensive analysis of all types of SVs in a cancer cell line and a tumor sample. Of note, sensitivity was here analysed with regard to all identified and confirmed SVs. However, true sensitivity may be lower, because additional SVs might exist that are neither detected by cWGS nor 10XWGS sequencing.

The reduced sensitivity in 10XWGS data raised a question whether it was a limitation of the analysis pipeline (ensemble of 10XWGS tools) or the technology did not cover the affected genomic regions. Interestingly, we observed that SVs, which were not identified by 10XWGS tools, did have support in the aligned linked-reads (i.e. overlapping GEM, JR and SP). We further analysed this by removal of barcodes in linked-reads and processed it with classical cWGS prediction tools. With the debarcoded sample, we were able to identify additional SVs that were missed by 10XWGS specific tools. This indicates that additional information is present in the raw 10XWGS sequencing data that is not fully utilized by currently available tools. Although the existing 10XWGS tools use similar category of evidence as cWGS tools (discordant read-pairs, split molecules, de novo assembly) apart from GEM coverage, they, however, seem to miss many true calls.

Previously, studies have shown that 10XWGS technology was especially useful in identifying complex genomic rearrangements or chained SVs [10]. Here we did not specifically address this subset of SVs, as we were interested in the overall performance of SV prediction. Nonetheless, the added benefit of 10XWGS sequencing becomes visible when looking at large SVs and translocations. This class of SVs is particularly difficult to resolve by the cWGS technology and suffers from high FDRs [36]. Utilization of long-range information by the 10XWGS pipeline should be powerful in resolving them. This was demonstrated by the fact that the 10XWGS pipeline reported a much lower and much more plausible number of translocations in comparison to the cWGS pipeline. We also observed for translocations the highest overlap (~48–53%) of the 10XWGS predictions with the cWGS pipeline that were all confirmed by PCR. However, only 65% of all high confidence translocations from the 10XWGS pipeline were confirmed by PCR. This suggests that not all translocations predicted by the 10XWGS pipeline are true events or are chained SVs. On the other hand, we were also able to confirm translocations reported only by the cWGS pipeline that were missed by the 10XWGS pipeline. Nevertheless, the 10XWGS pipeline was superior in predicting translocations in comparison to the cWGS pipeline.

The performance of cWGS technology suffers from high FDRs in low mappability and low complexity regions, such as simple repeats and LTRs [18], while the performance has previously been shown to be unaffected by SINE, LINE and DNA elements in the genome. In line with that, we identified a higher fraction of SVs in repetitive regions for cWGS technology compared to 10XWGS, especially in microsatellite, simple repeat and SINE elements. Furthermore, we observed a lower validation success rate for these SVs, demonstrating that a high fraction of predicted SVs in those regions are potentially false positive. Utilization of the long range information provided by 10XWGS seems to be able to greatly reduce these false positive predictions as indicated by a much smaller fraction of predicted SVs in those regions.

For both technologies we identified only a small fraction of SVs in regions with an ambiguous mapping of reads. Nonetheless, the fraction of SVs only identified with 10XWGS in such regions was more than double in comparison to cWGS. Moreover, 10XWGS technology did improve local coverage around breakpoints for SVs that were missed by cWGS pipeline. With the exception of translocations, all other type of large size SVs (size >10 kb) that were only identified by 10XWGS had significantly higher median local coverage around breakpoints from 10XWGS technology than cWGS. This indicated that the long range information utilized by 10XWGS allows improved mapping and coverage to those regions and improved subsequent identification of SVs.

Taken together, 10XWGS enabled more accurate detection of translocations and of SVs in low complexity regions. However, when all predicted SVs were considered, an improved detection on this subset does not translate into an overall improved FDR or sensitivity. This is also corroborated by other studies [10,33]. Our data had shown that this is largely due to methodology issues, demonstrating that the relatively new 10XWGS technology needs to catch up with methodological advancements from cWGS prediction tools.

Previous studies have also used a combination of cWGS and 10XWGS to predict SVs where 10XWGS data was often used as an orthogonal validation set. Confirming SVs predicted from cWGS technology with 10XWGS technology would lead to highly specific SVs, as we could confirm here by PCR. However, this comes also at the cost of missing a considerable fraction of true events.

Here we proposed an integrated statistical approach using both the technologies to achieve optimized FDR and sensitivity for all types of SVs. We tested the combined model on an exhaustive set of internally validated SVs and two externally validated data sets. We observed lower FDRs in comparison to FDRs of both technologies, however at the cost of minimal loss in sensitivity. The model efficiently combined different features as requantification, GEM support, type and size of SVs and local coverage around breakpoints. However, one limitation of this model would be for application in detection of chained SVs. Those events would have partial or no support from requantification pipeline. Nevertheless, it outperforms other tools for simple SVs and even a simple heuristic filter for the read support. We could also show the robustness of model with downsampled and debarcoded reads.

Another limitation of such an integrated approach is the requirement to run two sequencing experiments for each sample. Therefore, we generated models based on 10XWGS and cWGS pipeline independently. The overall performance of model was superior compared to the individual tools for the respective technologies. The individual models for cWGS and 10XWGS enables their usage when only one technology is available. This is of particular relevance for the 10XWGS data as our model provides a very prominent improvement in performance compared to the three tested 10XWGS tools. However, without cWGS data, a gap in sensitivity is evident. The debarcoding of 10XWGS data and its subsequent analysis with cWGS pipeline could provide an opportunity to boost sensitivity to almost the same level.

We also investigated shared germline SVs present in the gnomAD database. The fraction of MCF7 SVs present in gnomAD was low. However, individual or low frequency germline SVs of the respective samples are not covered by this analysis. Only the analysis of a matched sample would enable clear separation of germline and somatic SVs. Nonetheless we observed best sensitivity for known germline SVs with the combined model, indicating that these can be predicted with similar high sensitivity.

The sensitivities observed in our internally validated data set and existing datasets confirms this claim. Convincingly, the hereby used logistic regression approach with unique set of features opens up a broader application of the model.

Conclusively, our analysis for true SV events could show that specific and sensitive prediction of SVs is possible, but requires an integrative approach for best results. We could show that 10XWGS predicted SVs could be used for orthogonal validation but considering only those calls would miss many true events. Our combined model approach takes into account all the available data points to maintain high sensitivity and low FDR. Sensitive identification of SVs is necessary to get a complete picture of the mutational landscape in cancer and gain a better understanding of the disease. Additionally, the complex nature of many hereditary and genetic diseases could be resolved with reliable and sensitive prediction of SVs. Thus, we believe that the presented integrated prediction approach is a valuable tool that may identify novel targets for disease treatment.

## Supporting information

S1 Fig Workflow for calculation of requantification support with short-reads and GEM coverage for SVs. (A) Workflow to requantify supporting short-reads for SV. The reference genome sequence around the breakpoints A and B are extracted and fused according to the type and orientation of SVs. The short-reads are aligned to this fused genomic template. Junction reads (JR) and Spanning pairs (SP) are counted as requantification support. (B) Workflow to quantify unique GEMs or barcodes containing read-pairs that support a particular type and orientation of SV. First, discordant read pairs or split reads are retrieved from the 10XWGS pipeline generated alignment file. Then, unique GEMs are counted that support a particular SV type and orientation with breakpoints in window wi.
(TIF)

S2 Fig Construction of synthetic genomic template from the reference genome for calculation of requantification support. Illustration of the procedure to extract the reference genome sequence around the SV breakpoints that are fused to generate 1kb genomic templates. The fusion of genomic sequence around the breakpoints of SVs is performed according to the type of SV and the respective orientation (deletion-3’to5’, duplication-5’to3’, inversion fusion1-3’to3’, inversion fusion2-5’to5’). The same strategy is followed for translocation with the difference that the regions extracted belong to different chromosomes.
(TIF)

S3 Fig PCR primer design for different types of SVs.The left primer (LP) and right primer (RP) were designed at least 100bp up- and downstream the predicted breakpoints and were designed based upon the amplicon template formed according to the structural variation (deletion, duplication, inversion, translocation) and its orientation (3’to5’, 5’to3’, 3’to3’ and 5’to5’). (TIF)

S4 Fig SV type, sizes distribution of SVs predicted by cWGS and 10XWGS technology and percentage of common SVs amongst them. (A), (B) Number of different type of SVs predicted by two technologies in MCF7 and Primary tumor respectively. (C), (D) Percentage of high and low confidence calls overlapping between technologies for MCF7 and Primary tumor respectively. (E), (F) Distribution of size of different type of SVs from both the technologies in MCF7 Primary tumor respectively. (G), (H) Percentage of different SV types predicted by both the technologies in MCF7 and Primary tumor respectively.
(TIF)

S5 Fig Distribution of all SV calls from all cWGS and 10XWGS tools and their overlap, in MCF7 sequenced sample. (A) SV calls for all deletion, (B) duplication, (C) inversion, and (D) translocation. Low confidence calls are marked by “LowQ” and high confidence calls are marked by “PASS”.
(TIF)

S6 Fig Distribution of all SV calls from all cWGS and 10XWGS tools and their overlap, in Primary tumor. (A) SV calls for all deletions, (B) duplications, (C) inversions, and (D) translocations. Low confidence calls are marked by “LowQ” and high confidence calls are marked by “PASS”.
(TIF)

S7 Fig Distribution of SV calls (both high and low confidence) from cWGS sequenced MCF7, 10XWGS sequenced MCF7, downsampled cWGS reads in MCF7 to equivalent coverage as 10XWGS MCF7 (downsampled cWGS), removal of barcodes in 10XWGS linked-reads and processing them through cWGS tools (debarcoded 10XWGS). (A) The size distribution of different SV types for all mentioned samples, (B) Number of calls commonly predicted by 10XWGS, sequenced cWGS and downsampled cWGS; and number of calls commonly predicted by 10XWGS, sequenced cWGS and debarcoded 10XWGS (NOTE: Some of the SV calls from sequenced cWGS overlaps with multiple debarcoded 10XWGS and downsampled cWGS calls), (C) Number of SV calls processed from all mentioned samples (considering all SVs except duplications and inversions of size>10kb).
(TIF)

S8 Fig Only a small fraction of SVs overlap between the 10XWGS and cWGS predictions. (A), (B) Percentage of high and low confidence SVs from cWGS and 10XWGS pipeline that are common between technologies, in MCF7 and Primary tumor respectively. (C), (D) Percentage of 1 tool, 2 tools, 3 tools SVs from cWGS and 10XWGS pipeline common between the technologies, in MCF7 and Primay tumor respectively. (E) Number and percentage of common SV between two technologies that are predicted by 1 tool, 2 tools and 3 tools.
(TIF)

S9 Fig Common SVs have significantly higher support in terms of requantification (Sample = MCF7). Different requantification support (junction reads-JR, spanning pairs-SP, JR+SP = JRS) and GEM count plotted for common SVs, only cWGS SVs and only 10XWGS SVs. The requantification support was calculated from two sources of reads (cWGS and 10XWGS). p-value calculated with Kruskal-wallis test for comparison of three categories and pairwise Wilcoxon rank sum test. **** represents p-value <0.0001.
(TIF)

S10 Fig Common SVs have significantly higher support in terms of requantification (Sample = Primary tumor). Different requantification support (junction reads-JR, spanning pairs-SP, JR+SP = JRS) and GEM count plotted for common SVs, only cWGS SVs and only 10XWGS SVs. The requantification support was calculated from two sources of reads (cWGS and 10XWGS). p-value calculated with Kruskal-wallis test for comparison of three categories and pairwise Wilcoxon rank sum test. **** represents p-value <0.0001.
(TIF)

S11 Fig Requantification support and GEM count is higher for common SVs for different types of SVs (Sample = MCF7) for all calls or only high-confidence calls. The plot of three categories of SVs (common, only cWGS and only 10XWGS) and different type of SVs with respect to requantification support and GEM count. Requantification count was calculated from cWGS reads and 10XWGS reads separately. Junction reads (JR), spanning pairs (SP), combined support (JRS = JR+SP). ‘N’ represents the total number of SVs in the particular category.
(TIF)

S12 Fig Requantification support and GEM count is higher for common SVs for different types of SVs (Sample = Primary Tumor) for all calls or only high-confidence calls. The plot of three categories of SVs (common, only cWGS and only 10XWGS) and different type of SVs with respect to requantification support and GEM count. Requantification count was calculated from cWGS reads and 10XWGS reads separately. Junction reads (JR), spanning pairs (SP), combined support (JRS = JR+SP). ‘N’ represents the total number of SVs in the particular category.
(TIF)

S13 Fig Annotation of breakpoints of SVs shared between technologies indicate the advantage of each technology. (A) Breakpoints of all SVs (both high and low confidence) annotated with repetitive regions and their percentage across categories of SVs: common, only cWGS and only 10XWGS SVs. (B) Breakpoints of only high confidence SVs annotated with repetitive regions and their percentage across common SVs, only cWGS SVs and only 10XWGS SVs. (C) Breakpoints of all the SVs (both high and low confidence calls) annotated with unique mappability regions. (D) Normalized local coverage across two positions of each SV event in cWGS and 10XWGS aligned reads. All these figures depict annotation of breakpoints in MCF7 sample.
(TIF)

S14 Fig Some of the SVs common between technologies were not validated by PCR as their breakpoints lie in repetitive region, poor mappability region or when the reference genome was not annotated (Sample = MCF7). The table describes the possible reason for common SV calls that were not validated by PCR. Alignment of cWGS reads against reference genome for some negatively validated common SVs are shown in the form of IGV images.
(TIF)

S15 Fig Validation rate for SVs shared between all tools is higher for cWGS (A, B & C) and 10XWGS technology (D, E & F)-Sample MCF7. (A) Ratio of PCR validated SVs from the cWGS technology that were predicted by 1, 2 or 3 tools. (B) Ratio of different type of SVs from cWGS technology validated by PCR. (C) Ratio of different type of SVs validated by PCR with respect to prediction by 1, 2 or 3 tools for the cWGS technology. (D) Ratio of PCR validated SVs from the cWGS technology that were predicted by 1, 2 or 3 tools. (E) Ratio of different type of SVs by the 10XWGS technology validated by PCR. (F) Ratio of different type of SVs validated by PCR with respect to prediction by 1, 2 or 3 tools for the cWGS technology.
(TIF)

S16 Fig PCR validated SVs have significantly higher GEM and requantification support. p-values were derived from Wilcoxon-rank sum test (TIF)

S17 Fig Training and testing logistic regression model for cWGS and 10XWGS on the test data set. (A) An unbalanced data set for training as number of PCR validated SVs are higher than negative class from the cWGS technology. (B) Percentage importance of each feature used in the training of cWGS model calculated using varImp function of caret package. (C) Performance of the cWGS trained model on test data with different type of sampling for balancing the training data. (D) Percentage of relative feature importance calculated with dominance analysis using the complete set of PCR validated SVs trained with features derived from cWGS technology. The statistical significance was calculated using two-tailed test corresponding to z-ratio. (E) A balanced data set for training a model for 10XWGS technology. (F) Percentage importance of each feature used in the training of 10XWGS model calculated using varImp function of caret package. (G) Performance of the 10XWGS trained model on test data. (H) Percentage of relative feature importance calculated using dominance analysis with complete set of PCR validated SVs trained with features derived from 10XWGS technology. The statistical test was calculated using two-tailed test corresponding to z-ratio. The significance levels are: p-value<0.001 ‘***’, p-value<0.01 ‘**’, p-value<0.05 ‘*’, p-value<0.1
(TIF)

S18 Fig SVs predictions by trained combined model from cWGS and 10XWGS SVs in primary tumor. (A) Number of SVs prediction by the cWGS technology and percentage predicted by applying combined model. (B) Number of SVs prediction by the 10XWGS technology and percentage predicted by applying combined model. (C) Numbers and percentage of SVs common between technologies before (light colour) and after (dark colour) applying respective trained models.
(TIF)

S19 Fig Majority of breakpoints of filtered SVs by model lie in Non-repetitive, SINE or LINE regions in MCF7. (A) The graph depicts percentage of breakpoints of SVs that lie in different repetitive regions. The SVs were filtered with the best trained combined model. (B) Breakpoints of SVs filtered by best trained combined model annotated with repetitive regions and their percentage across common, only cWGS and only 10XWGS SVs.
(TIF)

S20 Fig The performance of combined model on internally validated SVs, two external data sets and gnomAD data set (Sample = MCF7). Sensitivity of combined model and other tools on the four data sets where SVs were predicted from (A) sequenced MCF7 sample. (B) downsampled cWGS MCF7 (equivalent coverage to 10XWGS MCF7 sample). (C) debarcoded 10XWGS linked-reads and processed with cWGS pipeline (for MCF7). (D) Overall performance of combined model on internally validated SVs with SVs predicted from downsampled cWGS reads. (E) Overall performance of combined model on internally validated SVs with SVs predicted from debarcoded 10XWGS linked-reads and processed with cWGS pipeline (for MCF7). (F) Number of gnomAD calls also present in SV calls filtered by the combined model in sequenced MCF7 sample.
(TIF)

S1 Table Sequencing statistics for MCF7 and Primary tumor with both the technologies. (XLSX)

S2 Table PCR primers, PCR and Sanger sequencing results for SVs tested in MCF7. (CSV)

S3 Table Sensitivity and precision of combined model against other tools on external data set 1, 2 and gnomAD calls. (XLSX)
