# Supplementary figures and images for: Integrative analysis of structural variations using short-reads and linked-reads yields highly specific and sensitive predictions
Source: PLoS Comput Biol. 2020 Nov 23;16(11):e1008397. doi: 10.1371/journal.pcbi.1008397 (PMC7721175; doi:10.1371/journal.pcbi.1008397)

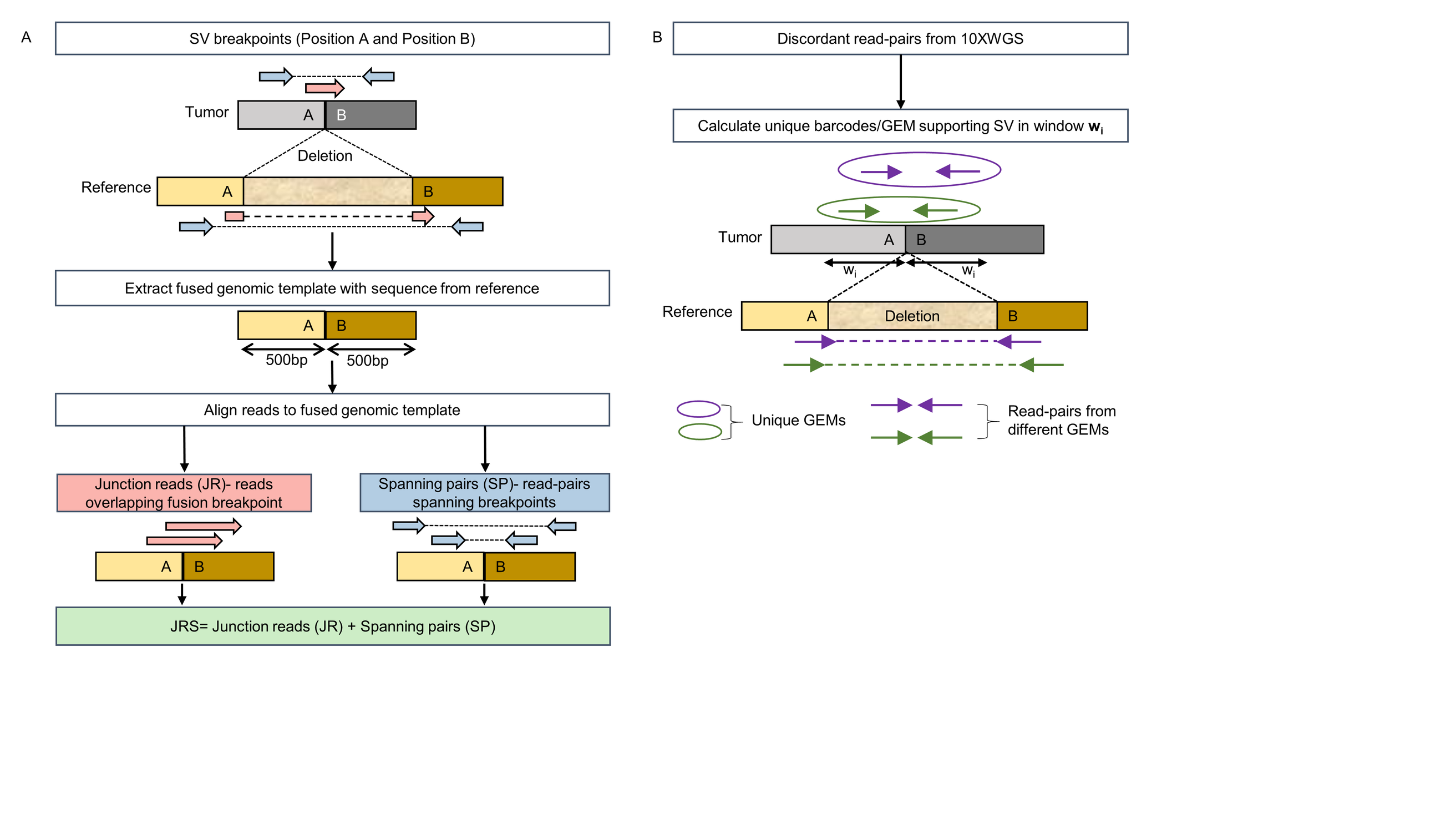

Supplement: S1 Fig — (A) Workflow to requantify supporting short-reads for SV. The reference genome sequence around the breakpoints A and B are extracted and fused according to the type and orientation of SVs. The short-reads are aligned to this fused genomic template. Junction reads (JR) and Spanning pairs (SP) are counted as requantification support. (B) Workflow to quantify unique GEMs or barcodes containing read-pairs that support a particular type and orientation of SV. First, discordant read pairs or split reads are retrieved from the 10XWGS pipeline generated alignment file. Then, unique GEMs are counted that support a particular SV type and orientation with breakpoints in window wi. (TIF) [file pcbi.1008397.s001.tif]

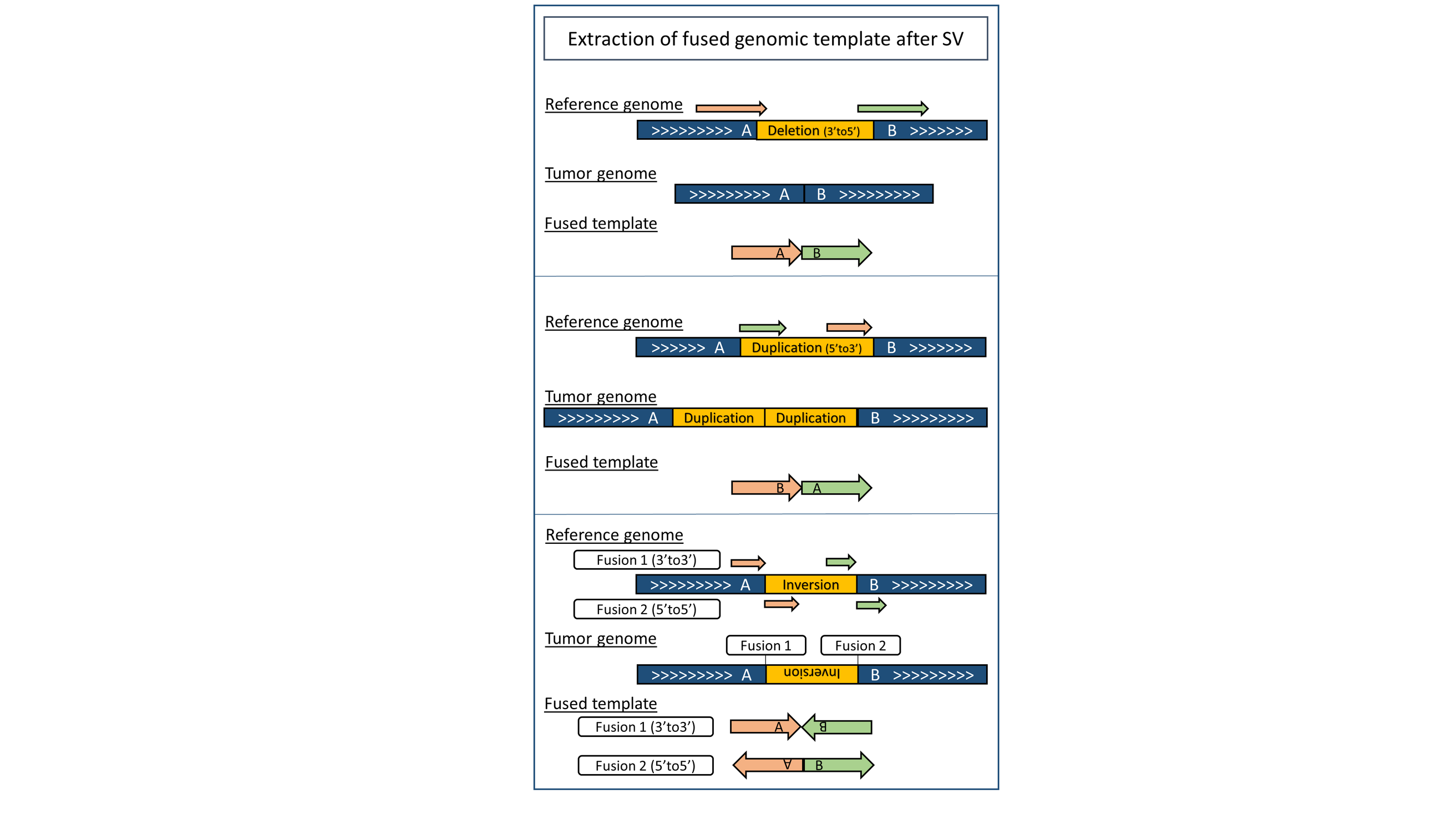

Supplement: S2 Fig — Illustration of the procedure to extract the reference genome sequence around the SV breakpoints that are fused to generate 1kb genomic templates. The fusion of genomic sequence around the breakpoints of SVs is performed according to the type of SV and the respective orientation (deletion-3’to5’, duplication-5’to3’, inversion fusion1-3’to3’, inversion fusion2-5’to5’). The same strategy is followed for translocation with the difference that the regions extracted belong to different chromosomes. (TIF) [file pcbi.1008397.s002.tif]

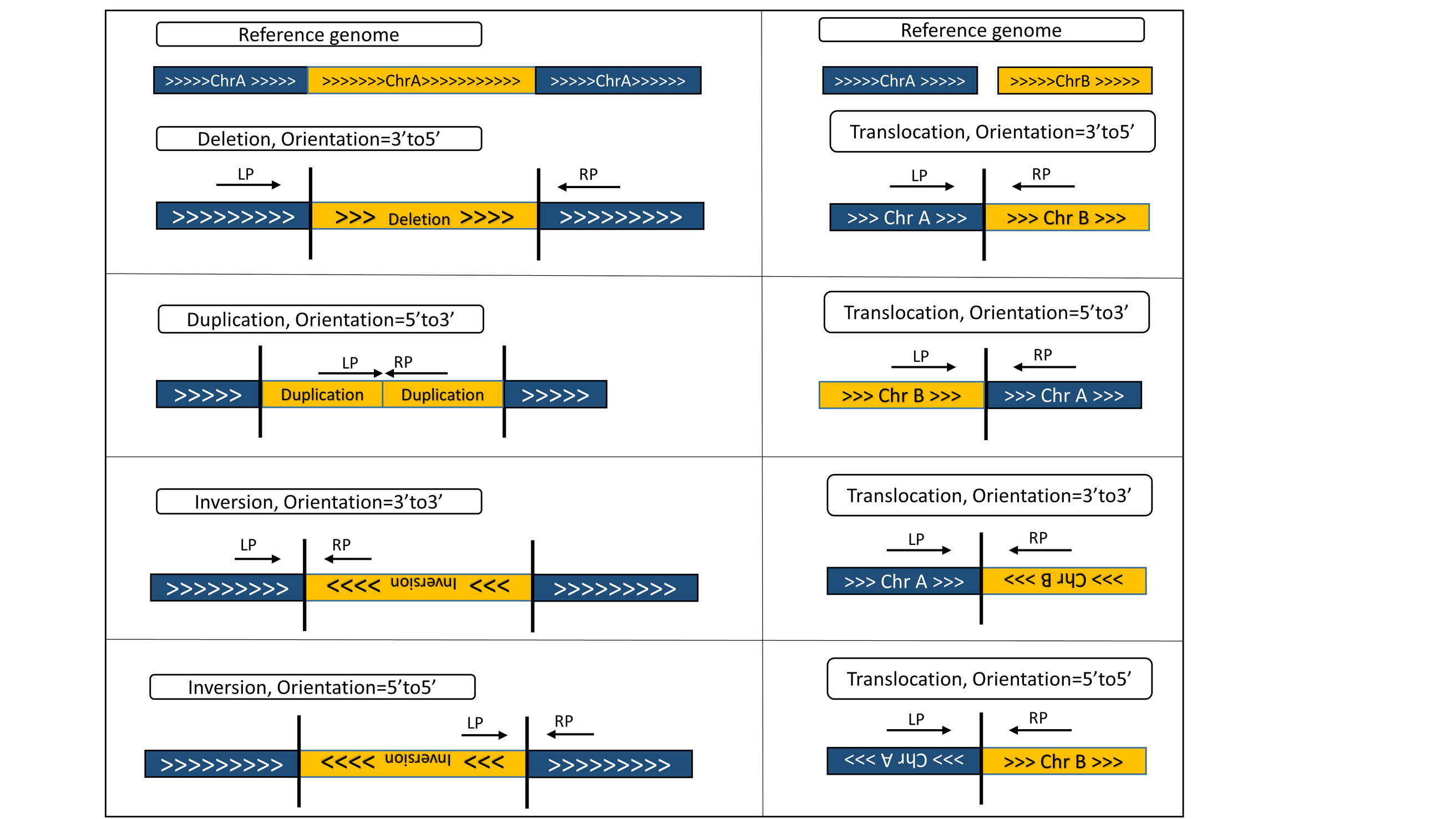

Supplement: S3 Fig — (TIF) [file pcbi.1008397.s003.tif]

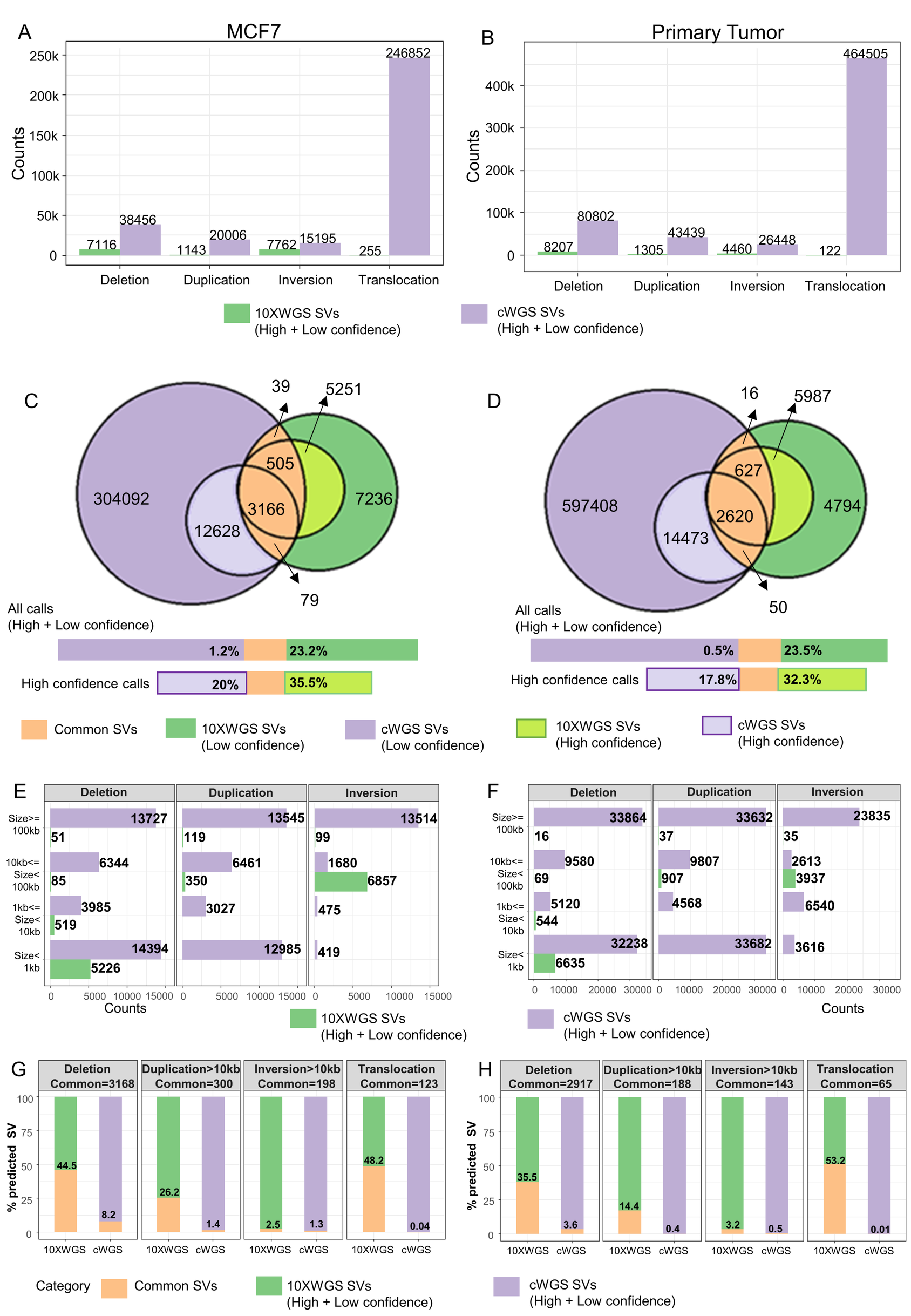

Supplement: S4 Fig — (A), (B) Number of different type of SVs predicted by two technologies in MCF7 and Primary tumor respectively. (C), (D) Percentage of high and low confidence calls overlapping between technologies for MCF7 and Primary tumor respectively. (E), (F) Distribution of size of different type of SVs from both the technologies in MCF7 Primary tumor respectively. (G), (H) Percentage of different SV types predicted by both the technologies in MCF7 and Primary tumor respectively. (TIF) [file pcbi.1008397.s004.tif]

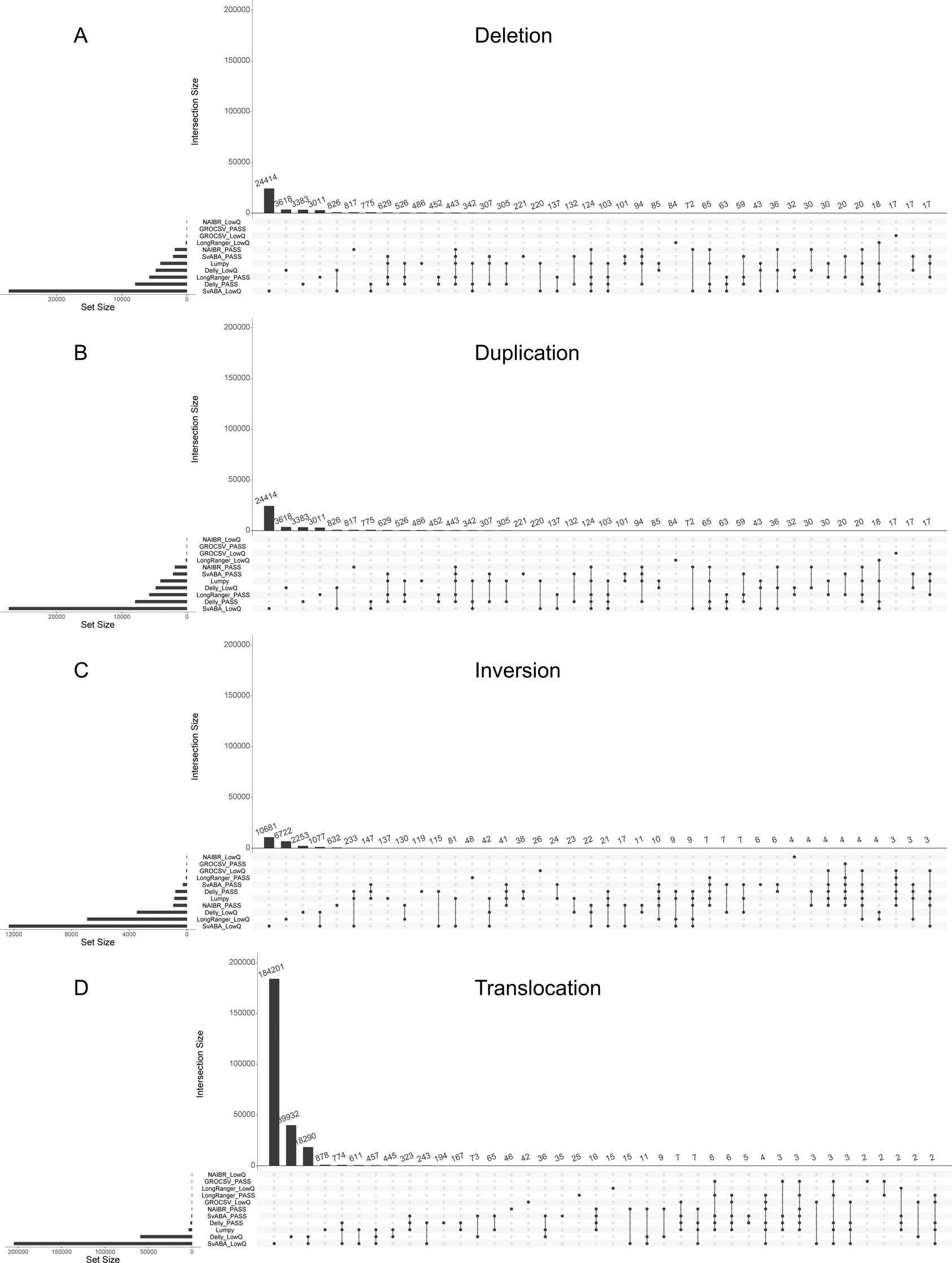

Supplement: S5 Fig — (A) SV calls for all deletion, (B) duplication, (C) inversion, and (D) translocation. Low confidence calls are marked by “LowQ” and high confidence calls are marked by “PASS”. (TIF) [file pcbi.1008397.s005.tif]

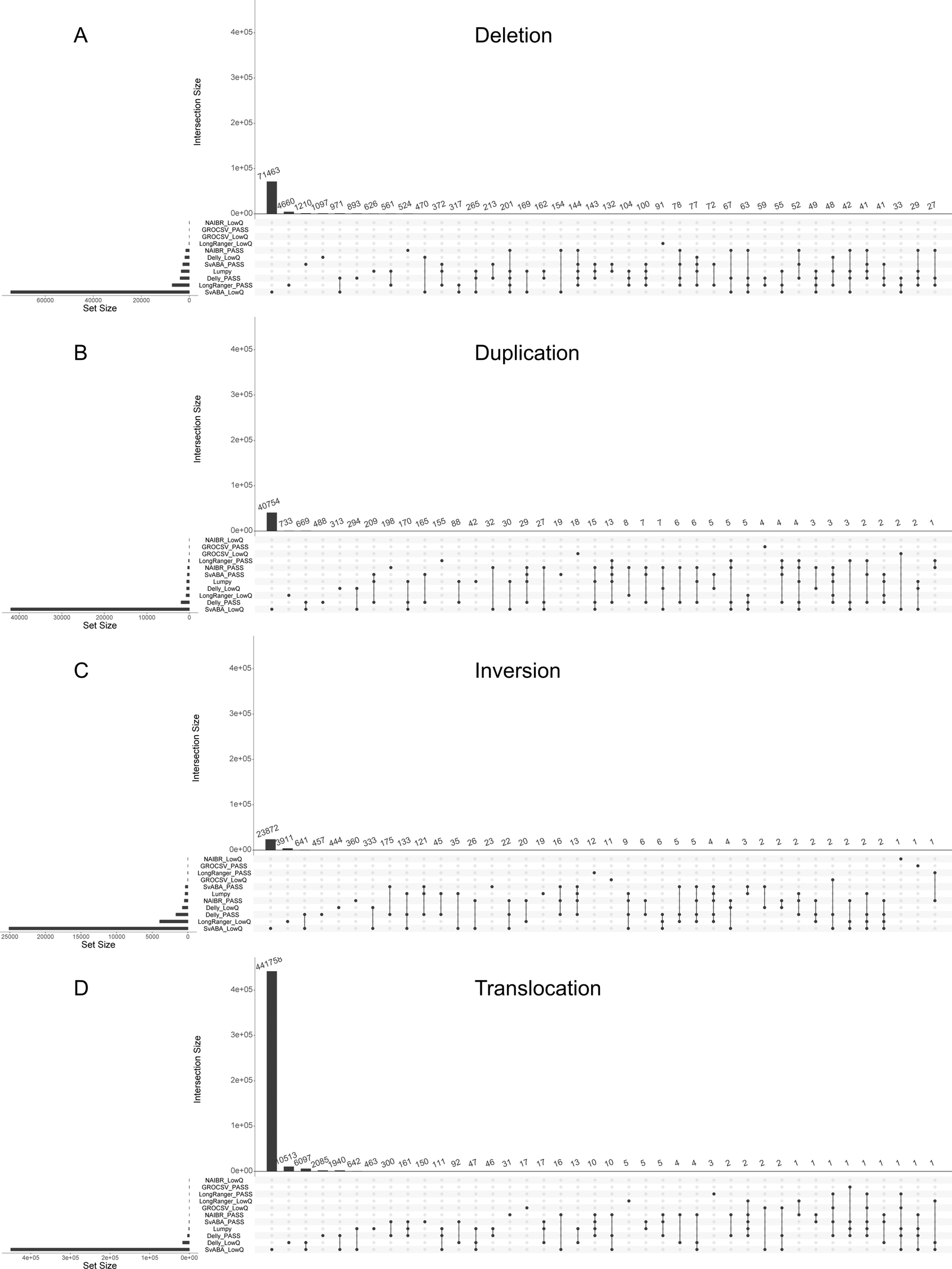

Supplement: S6 Fig — (A) SV calls for all deletions, (B) duplications, (C) inversions, and (D) translocations. Low confidence calls are marked by “LowQ” and high confidence calls are marked by “PASS”. (TIF) [file pcbi.1008397.s006.tif]

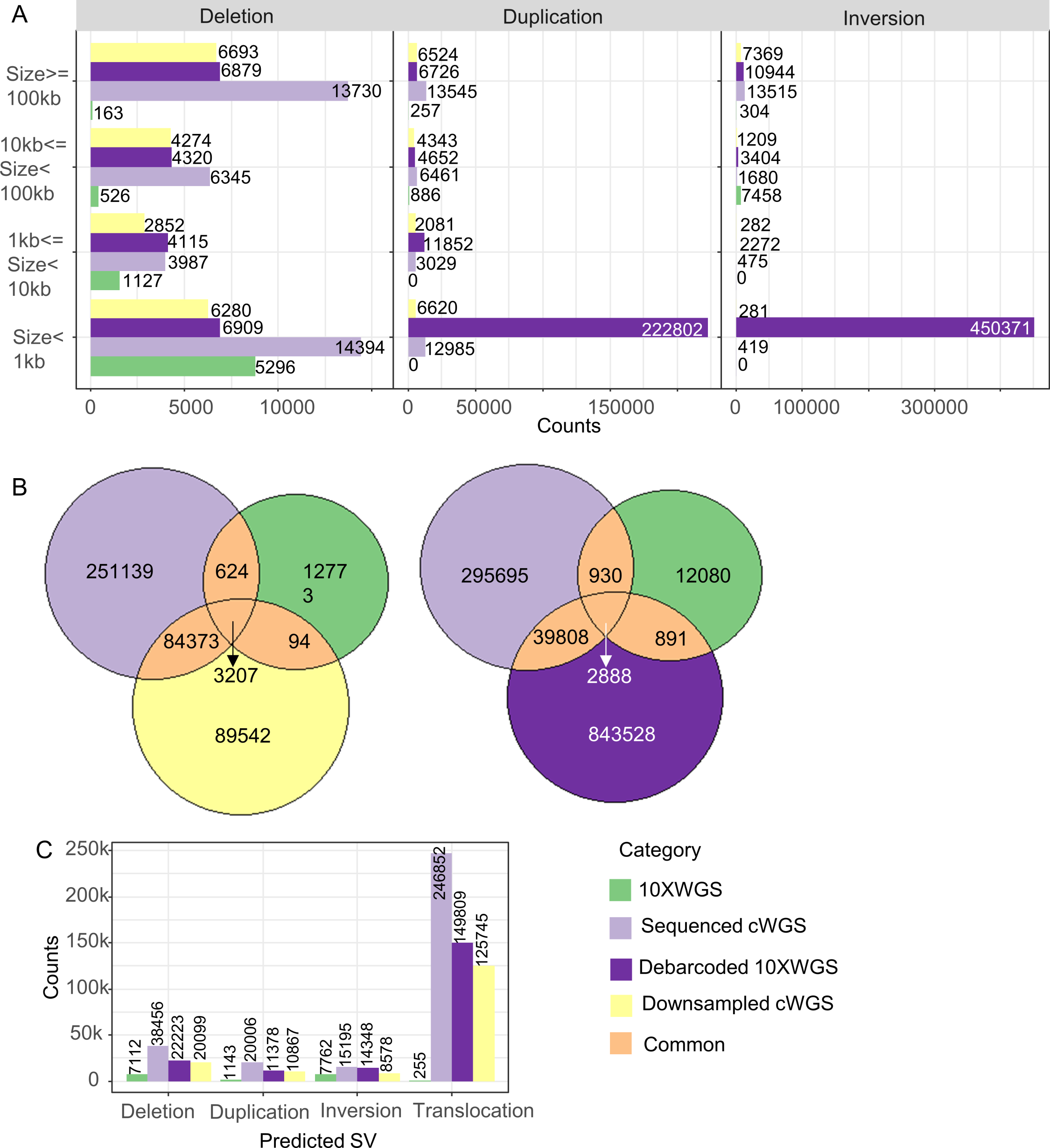

Supplement: S7 Fig — (A) The size distribution of different SV types for all mentioned samples, (B) Number of calls commonly predicted by 10XWGS, sequenced cWGS and downsampled cWGS; and number of calls commonly predicted by 10XWGS, sequenced cWGS and debarcoded 10XWGS (NOTE: Some of the SV calls from sequenced cWGS overlaps with multiple debarcoded 10XWGS and downsampled cWGS calls), (C) Number of SV calls processed from all mentioned samples (considering all SVs except duplications and inversions of size>10kb). (TIF) [file pcbi.1008397.s007.tif]

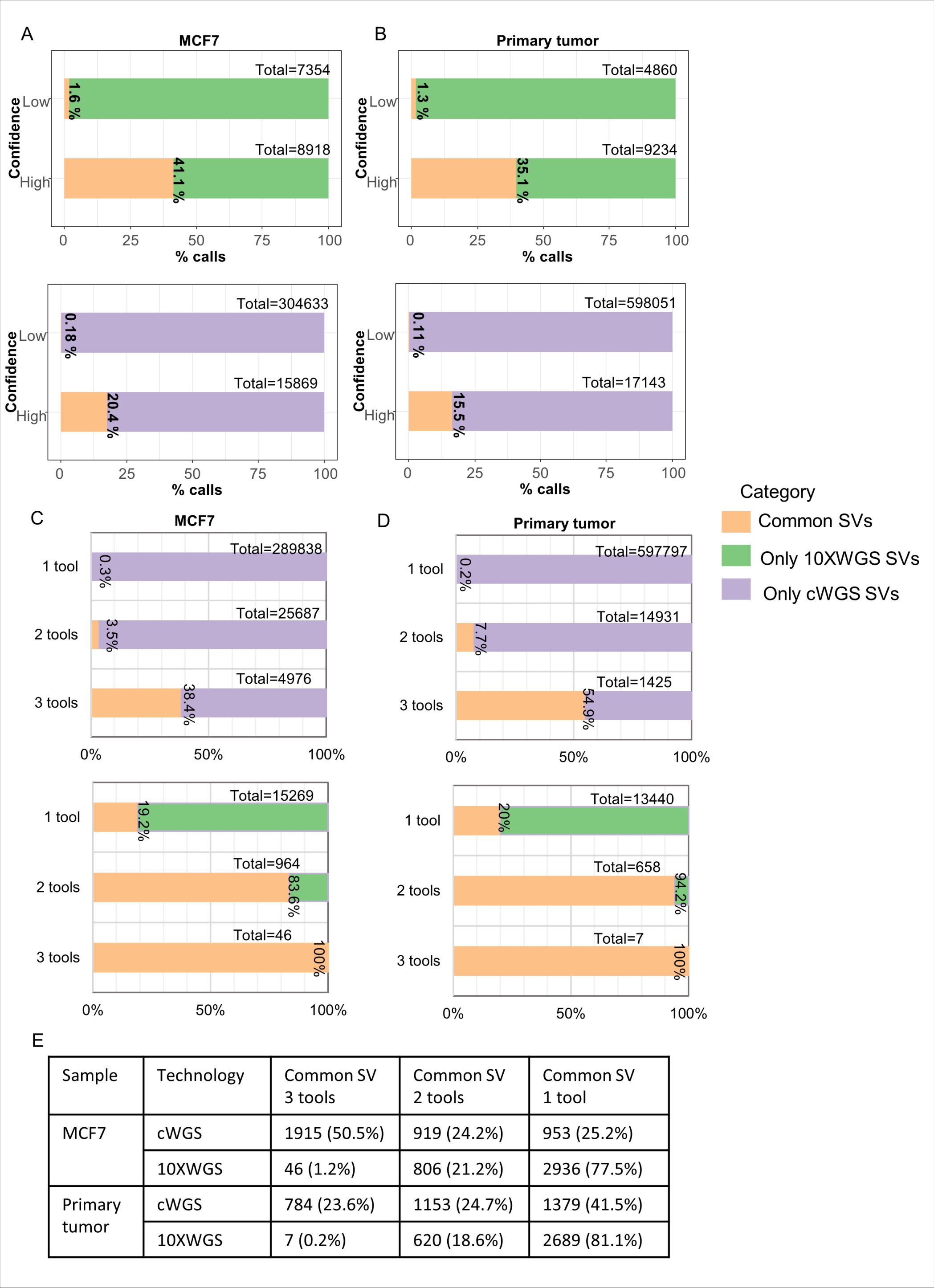

Supplement: S8 Fig — (A), (B) Percentage of high and low confidence SVs from cWGS and 10XWGS pipeline that are common between technologies, in MCF7 and Primary tumor respectively. (C), (D) Percentage of 1 tool, 2 tools, 3 tools SVs from cWGS and 10XWGS pipeline common between the technologies, in MCF7 and Primay tumor respectively. (E) Number and percentage of common SV between two technologies that are predicted by 1 tool, 2 tools and 3 tools. (TIF) [file pcbi.1008397.s008.tif]

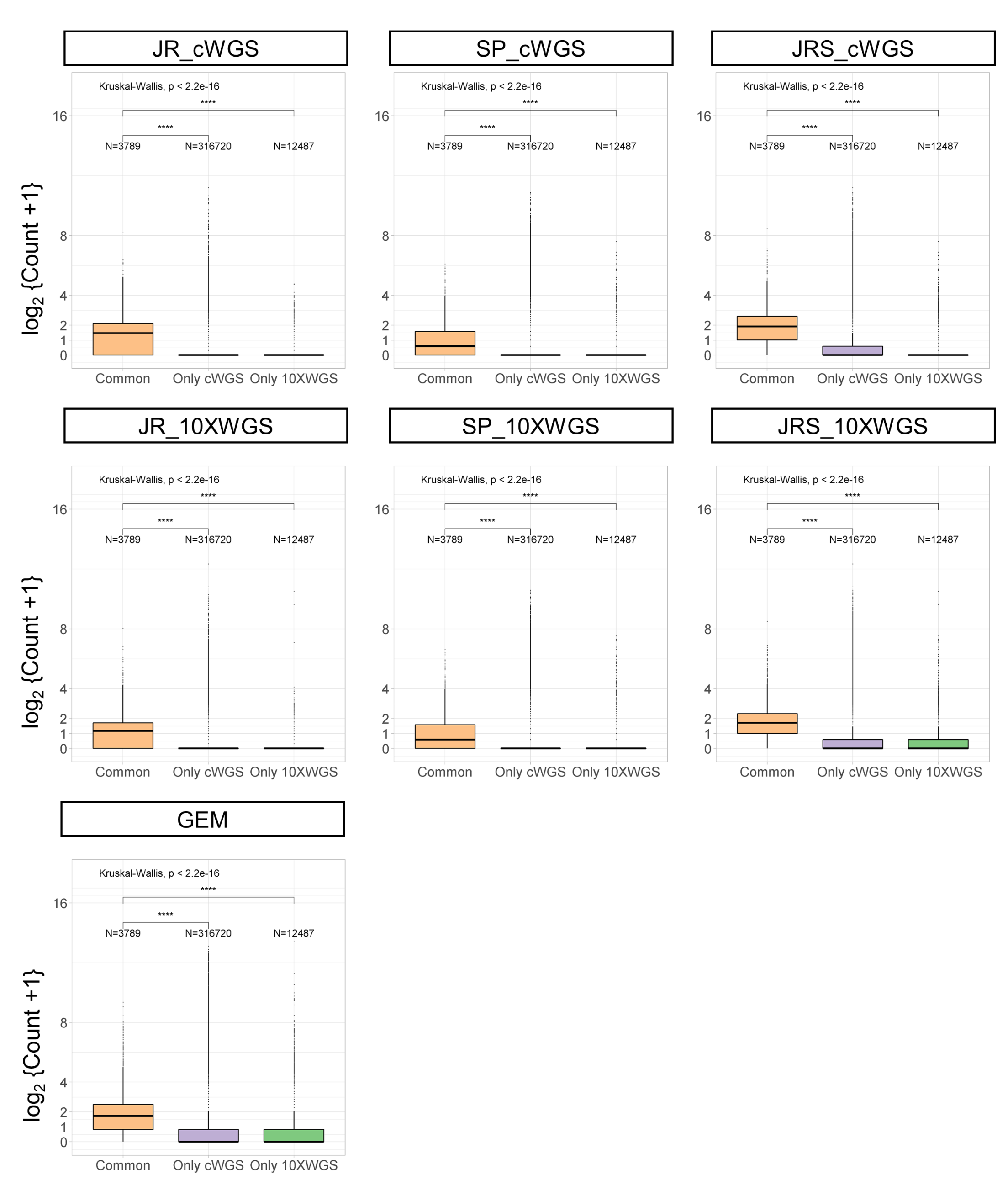

Supplement: S9 Fig — Different requantification support (junction reads-JR, spanning pairs-SP, JR+SP = JRS) and GEM count plotted for common SVs, only cWGS SVs and only 10XWGS SVs. The requantification support was calculated from two sources of reads (cWGS and 10XWGS). p-value calculated with Kruskal-wallis test for comparison of three categories and pairwise Wilcoxon rank sum test. **** represents p-value <0.0001. (TIF) [file pcbi.1008397.s009.tif]

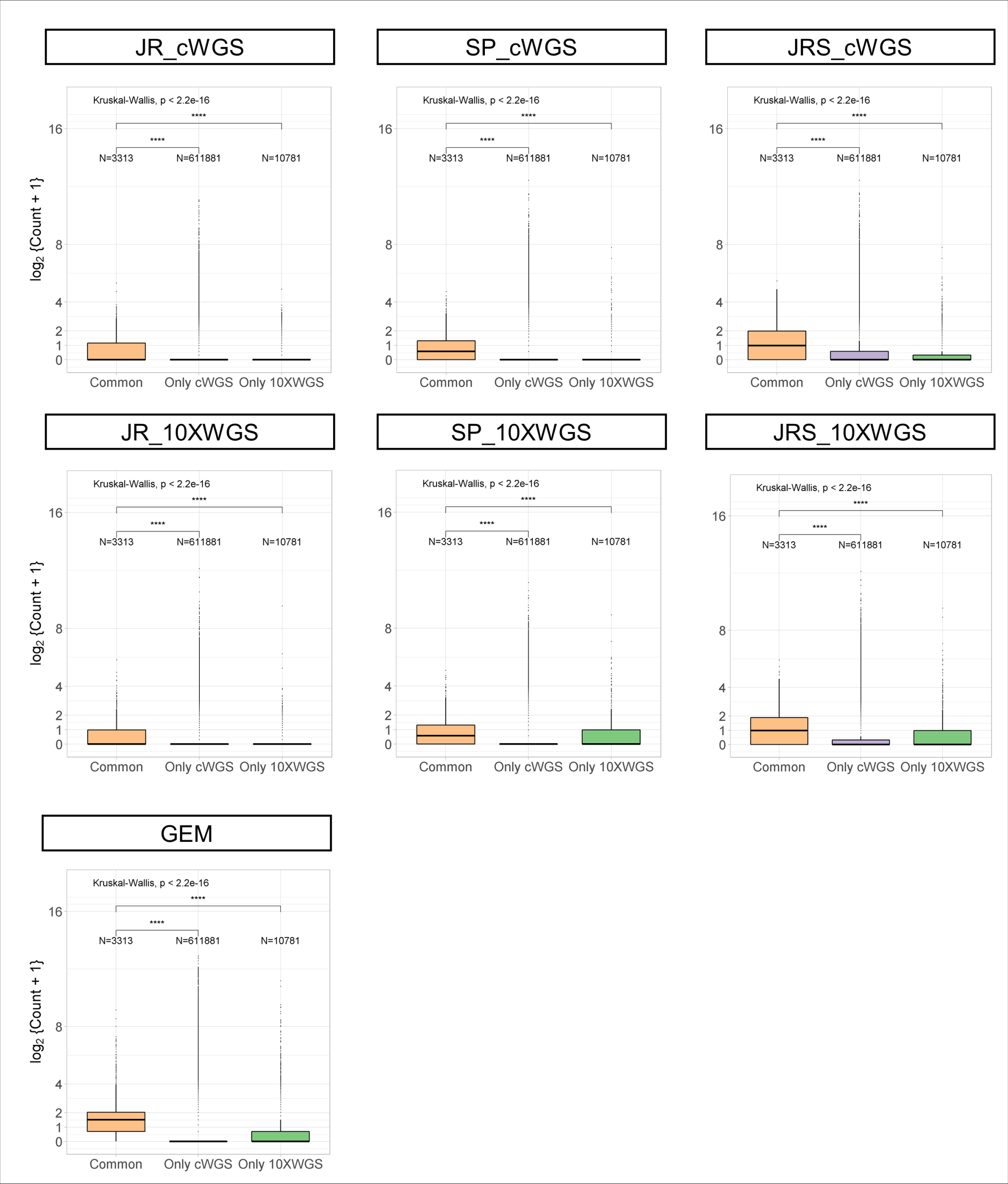

Supplement: S10 Fig — Different requantification support (junction reads-JR, spanning pairs-SP, JR+SP = JRS) and GEM count plotted for common SVs, only cWGS SVs and only 10XWGS SVs. The requantification support was calculated from two sources of reads (cWGS and 10XWGS). p-value calculated with Kruskal-wallis test for comparison of three categories and pairwise Wilcoxon rank sum test. **** represents p-value <0.0001. (TIF) [file pcbi.1008397.s010.tif]

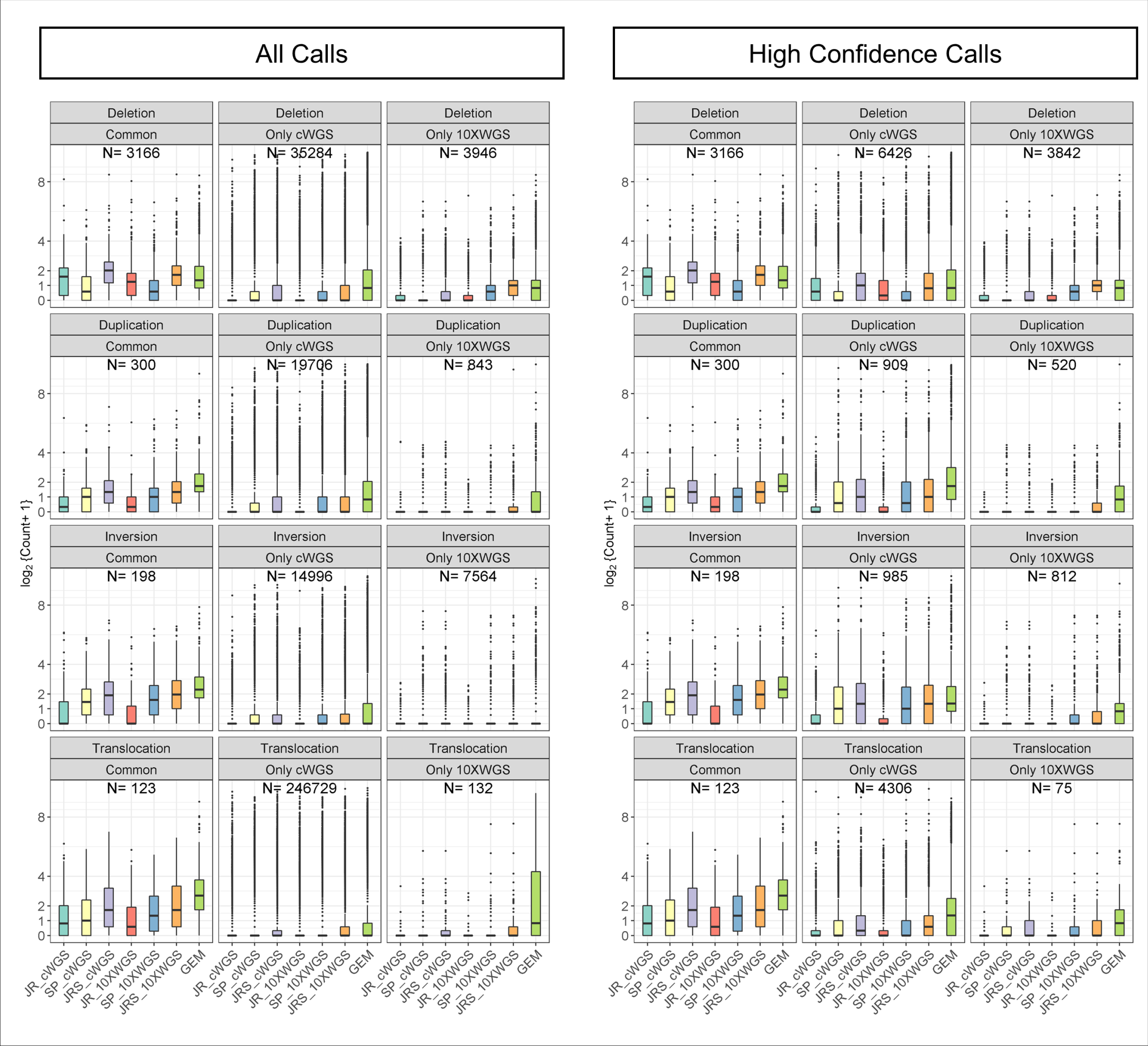

Supplement: S11 Fig — The plot of three categories of SVs (common, only cWGS and only 10XWGS) and different type of SVs with respect to requantification support and GEM count. Requantification count was calculated from cWGS reads and 10XWGS reads separately. Junction reads (JR), spanning pairs (SP), combined support (JRS = JR+SP). ‘N’ represents the total number of SVs in the particular category. (TIF) [file pcbi.1008397.s011.tif]

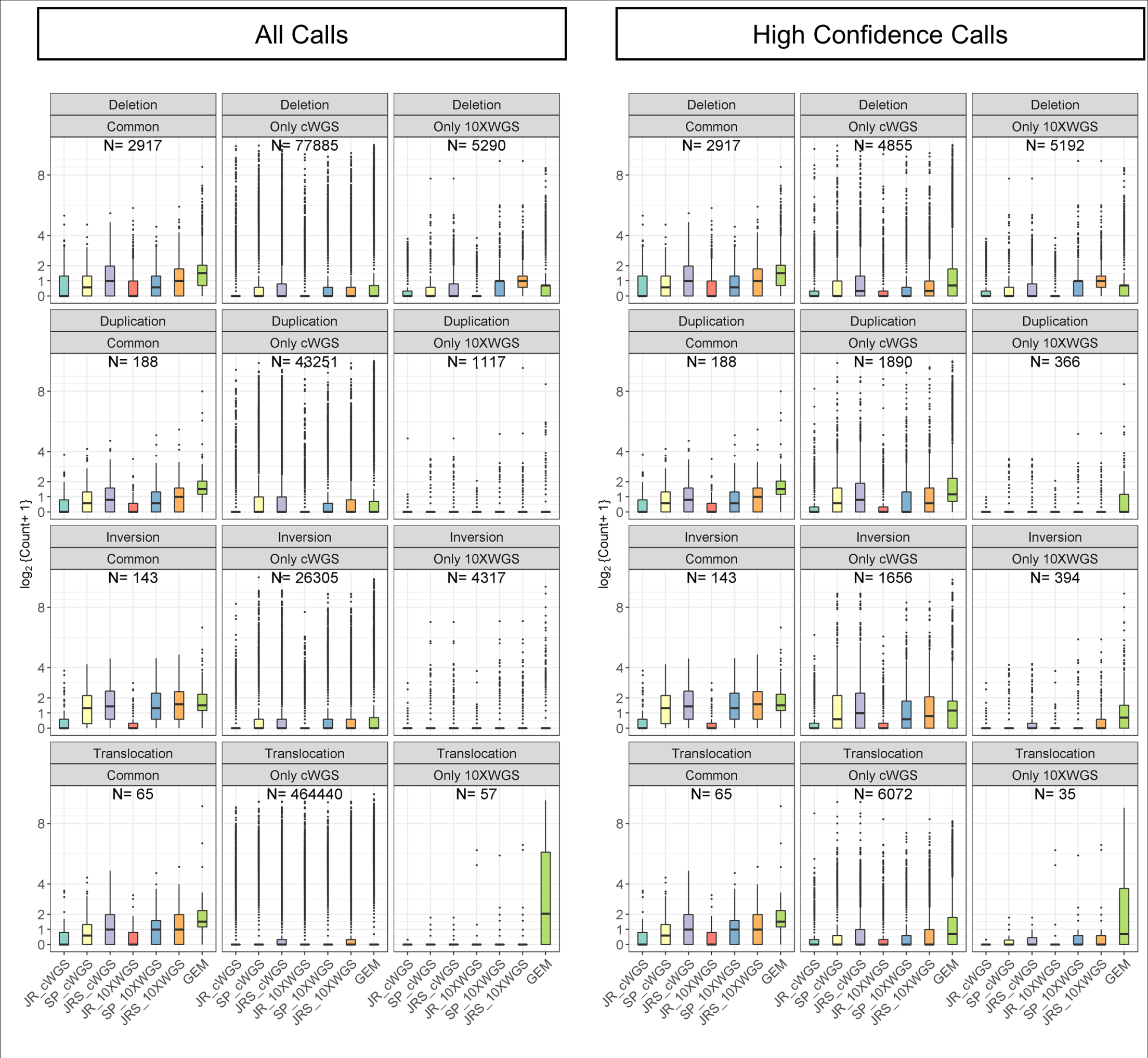

Supplement: S12 Fig — The plot of three categories of SVs (common, only cWGS and only 10XWGS) and different type of SVs with respect to requantification support and GEM count. Requantification count was calculated from cWGS reads and 10XWGS reads separately. Junction reads (JR), spanning pairs (SP), combined support (JRS = JR+SP). ‘N’ represents the total number of SVs in the particular category. (TIF) [file pcbi.1008397.s012.tif]

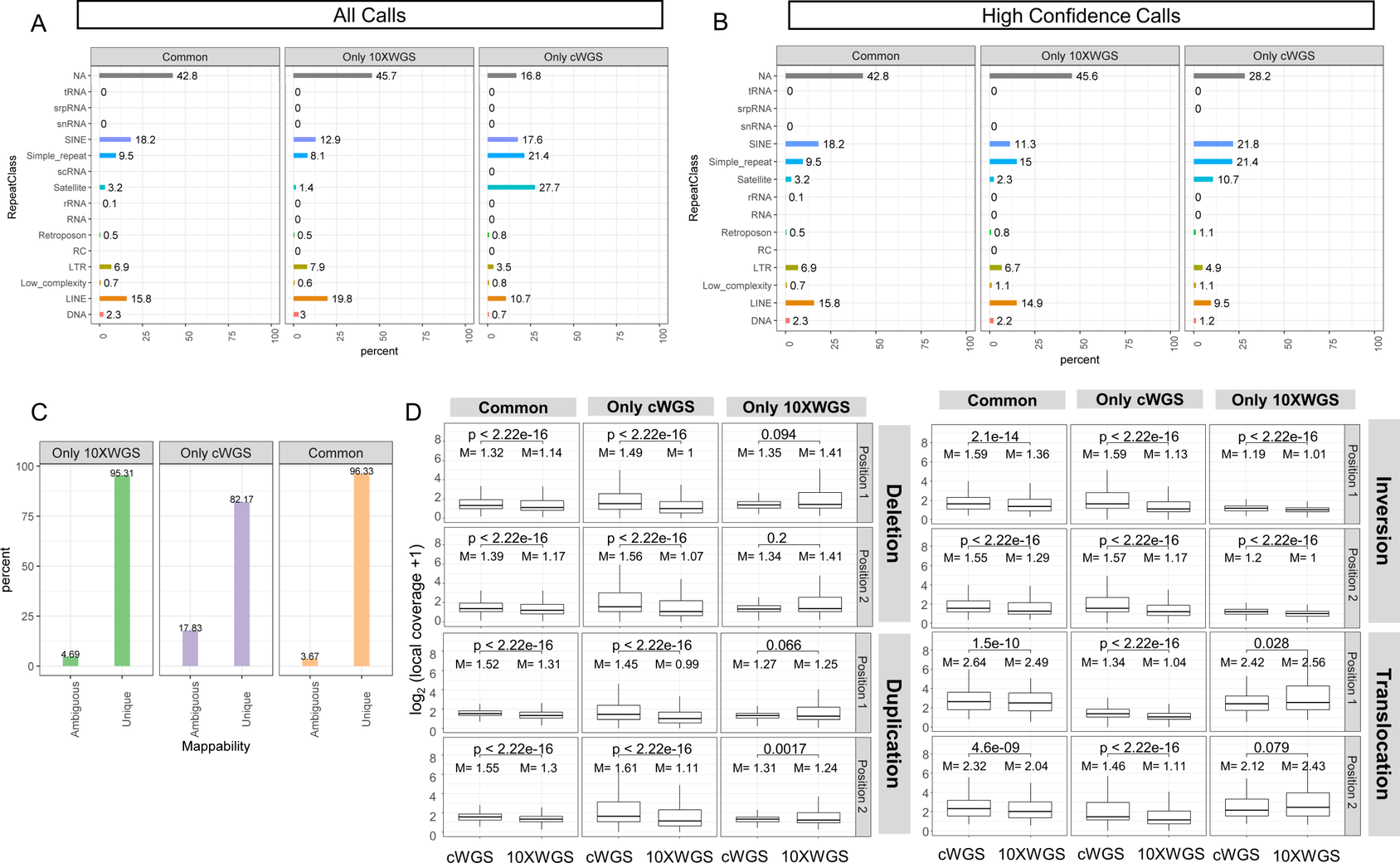

Supplement: S13 Fig — (A) Breakpoints of all SVs (both high and low confidence) annotated with repetitive regions and their percentage across categories of SVs: common, only cWGS and only 10XWGS SVs. (B) Breakpoints of only high confidence SVs annotated with repetitive regions and their percentage across common SVs, only cWGS SVs and only 10XWGS SVs. (C) Breakpoints of all the SVs (both high and low confidence calls) annotated with unique mappability regions. (D) Normalized local coverage across two positions of each SV event in cWGS and 10XWGS aligned reads. All these figures depict annotation of breakpoints in MCF7 sample. (TIF) [file pcbi.1008397.s013.tif]

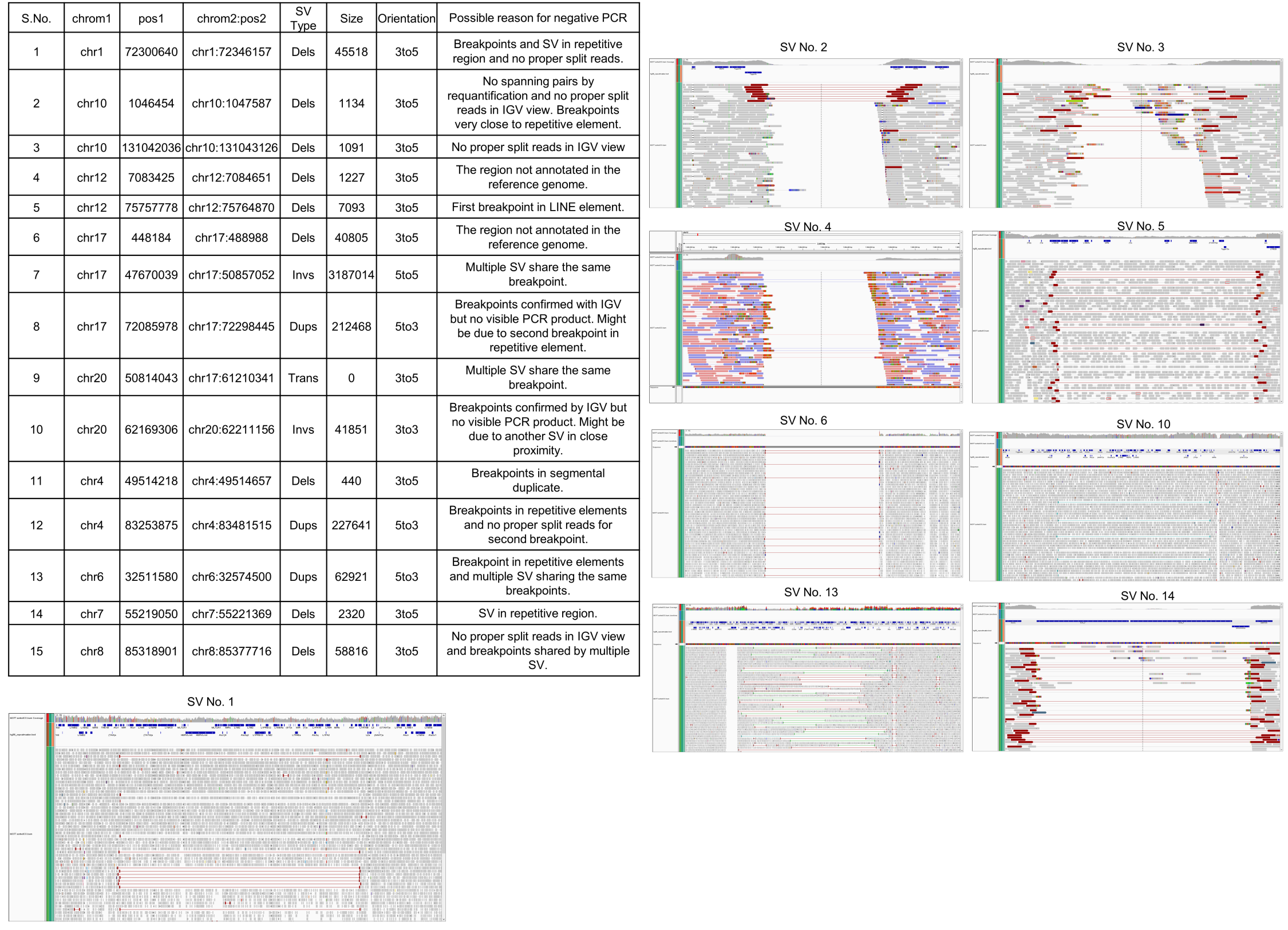

Supplement: S14 Fig — The table describes the possible reason for common SV calls that were not validated by PCR. Alignment of cWGS reads against reference genome for some negatively validated common SVs are shown in the form of IGV images. (TIF) [file pcbi.1008397.s014.tif]

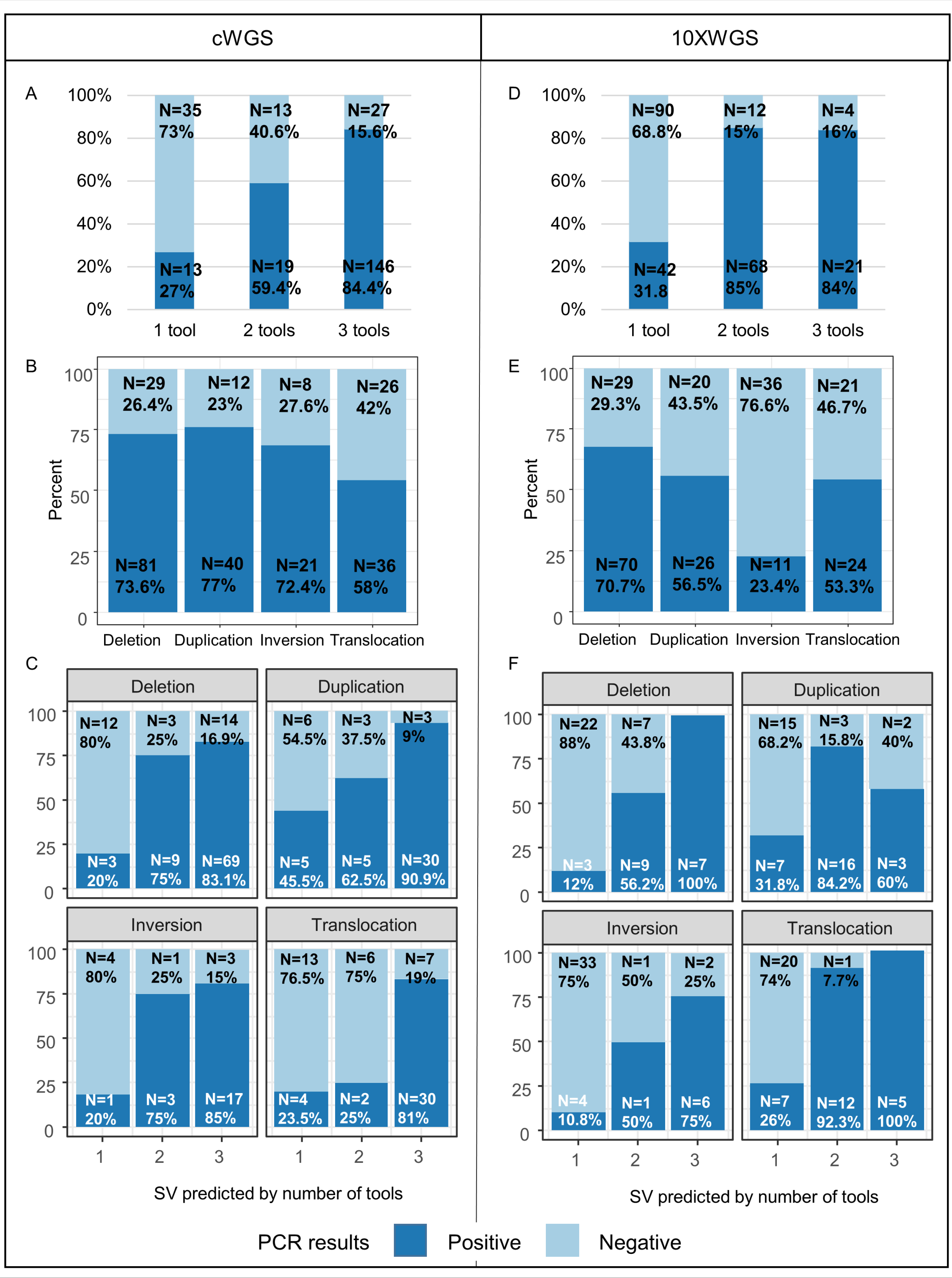

Supplement: S15 Fig — Validation rate for SVs shared between all tools is higher for cWGS (A, B & C) and 10XWGS technology (D, E & F)-Sample MCF7. (A) Ratio of PCR validated SVs from the cWGS technology that were predicted by 1, 2 or 3 tools. (B) Ratio of different type of SVs from cWGS technology validated by PCR. (C) Ratio of different type of SVs validated by PCR with respect to prediction by 1, 2 or 3 tools for the cWGS technology. (D) Ratio of PCR validated SVs from the cWGS technology that were predicted by 1, 2 or 3 tools. (E) Ratio of different type of SVs by the 10XWGS technology validated by PCR. (F) Ratio of different type of SVs validated by PCR with respect to prediction by 1, 2 or 3 tools for the cWGS technology. (TIF) [file pcbi.1008397.s015.tif]

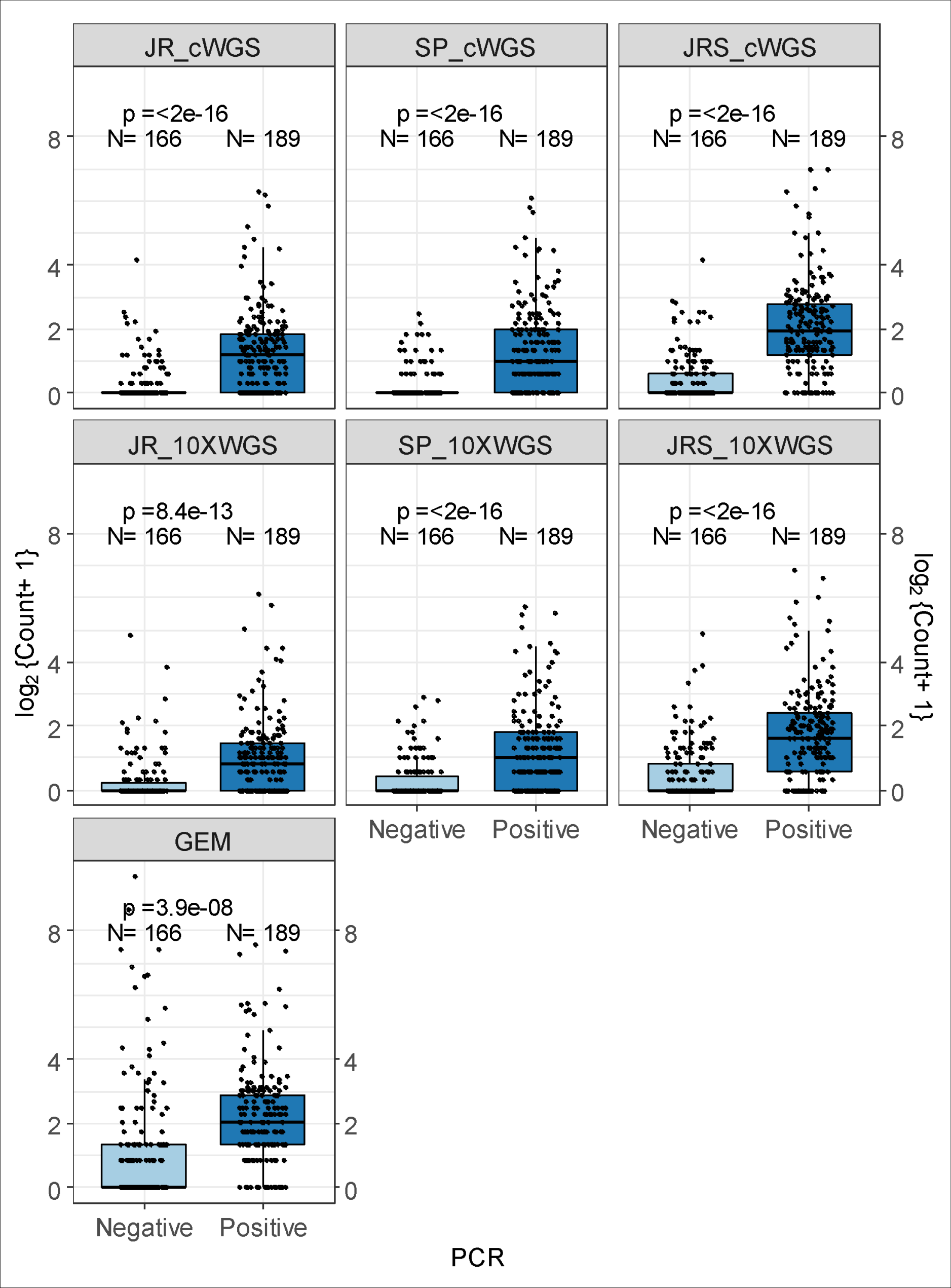

Supplement: S16 Fig — (TIF) [file pcbi.1008397.s016.tif]

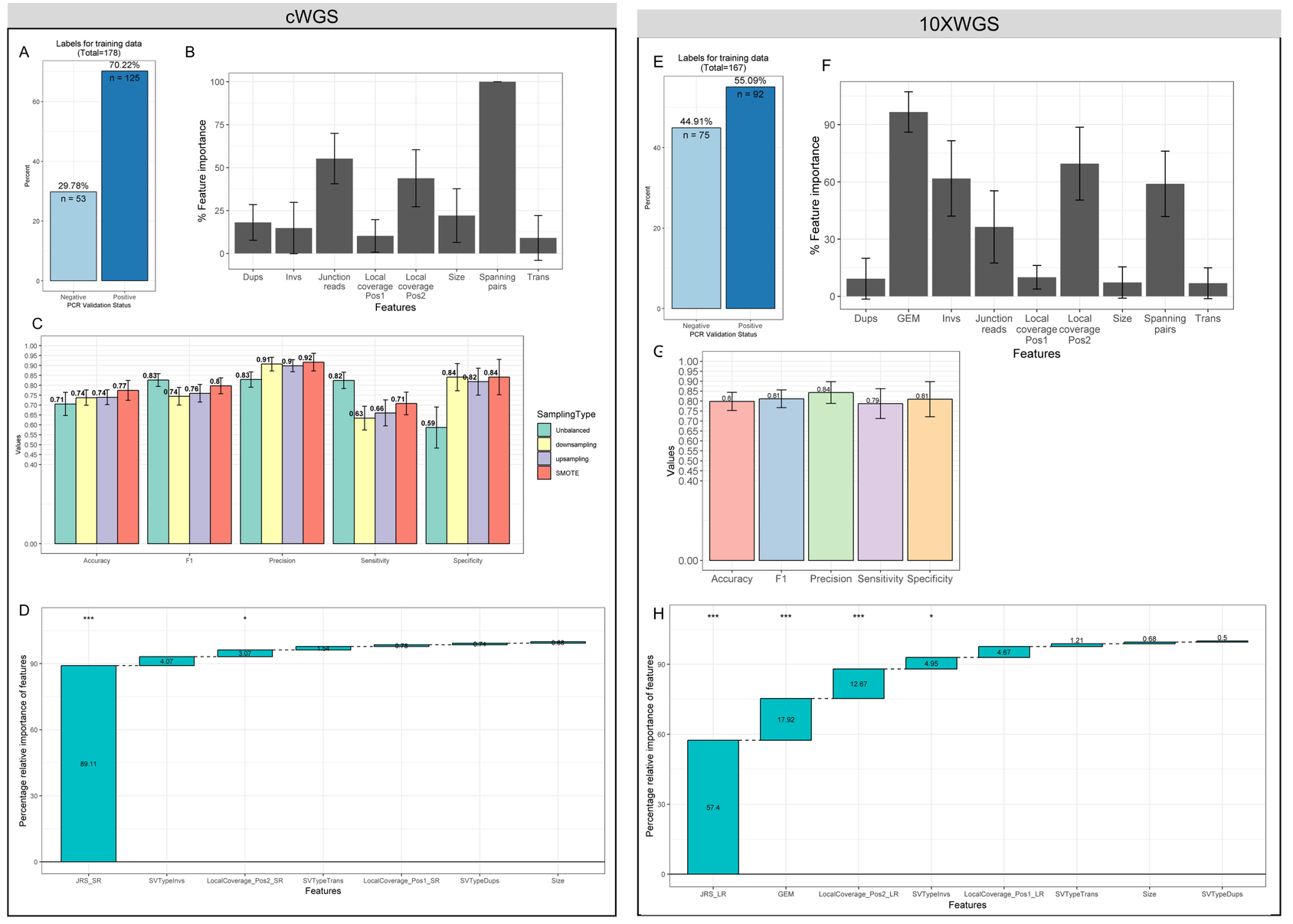

Supplement: S17 Fig — (A) An unbalanced data set for training as number of PCR validated SVs are higher than negative class from the cWGS technology. (B) Percentage importance of each feature used in the training of cWGS model calculated using varImp function of caret package. (C) Performance of the cWGS trained model on test data with different type of sampling for balancing the training data. (D) Percentage of relative feature importance calculated with dominance analysis using the complete set of PCR validated SVs trained with features derived from cWGS technology. The statistical significance was calculated using two-tailed test corresponding to z-ratio. (E) A balanced data set for training a model for 10XWGS technology. (F) Percentage importance of each feature used in the training of 10XWGS model calculated using varImp function of caret package. (G) Performance of the 10XWGS trained model on test data. (H) Percentage of relative feature importance calculated using dominance analysis with complete set of PCR validated SVs trained with features derived from 10XWGS technology. The statistical test was calculated using two-tailed test corresponding to z-ratio. The significance levels are: p-value<0.001 ‘***’, p-value<0.01 ‘**’, p-value<0.05 ‘*’, p-value<0.1 (TIF) [file pcbi.1008397.s017.tif]

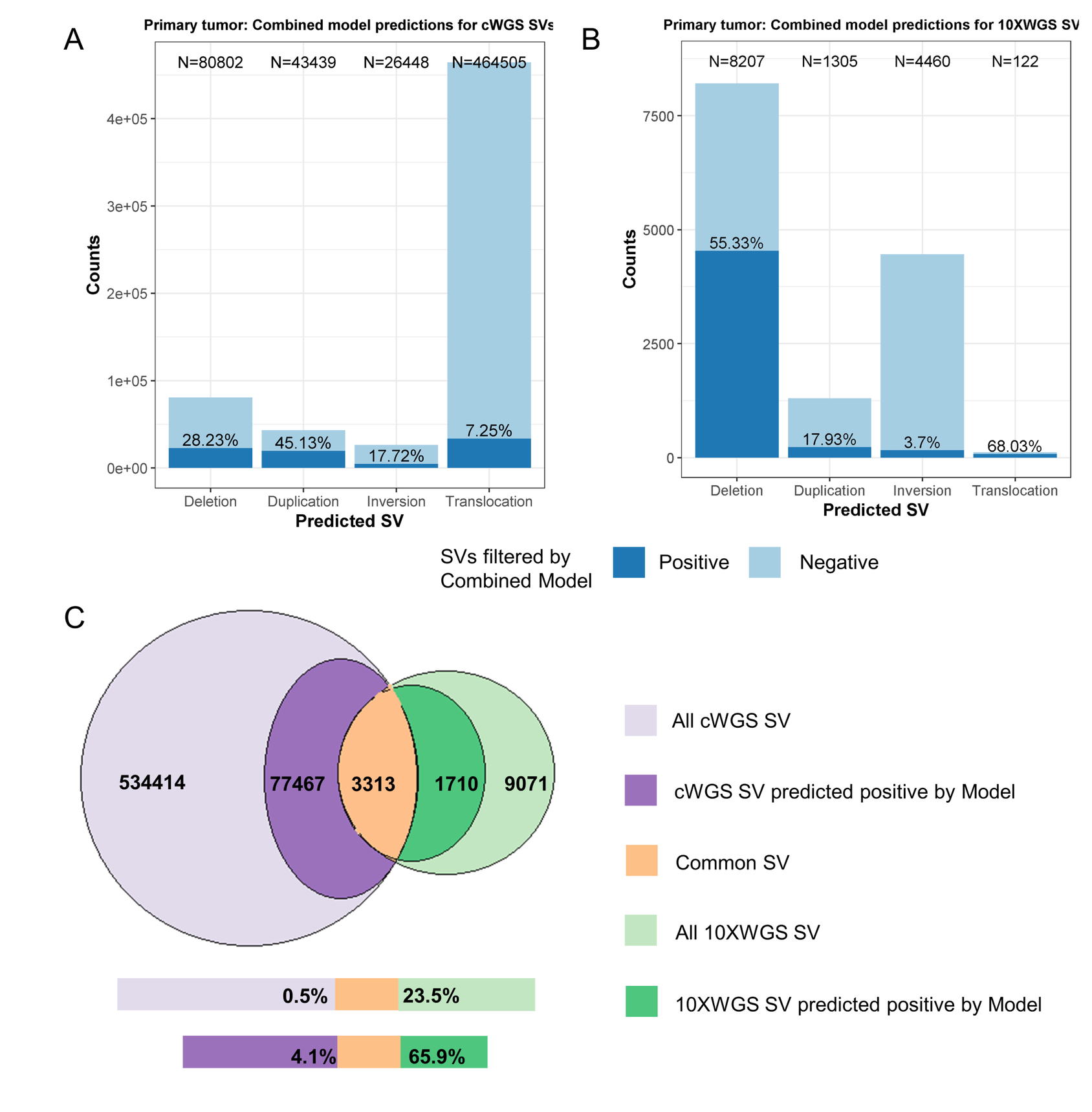

Supplement: S18 Fig — (A) Number of SVs prediction by the cWGS technology and percentage predicted by applying combined model. (B) Number of SVs prediction by the 10XWGS technology and percentage predicted by applying combined model. (C) Numbers and percentage of SVs common between technologies before (light colour) and after (dark colour) applying respective trained models. (TIF) [file pcbi.1008397.s018.tif]

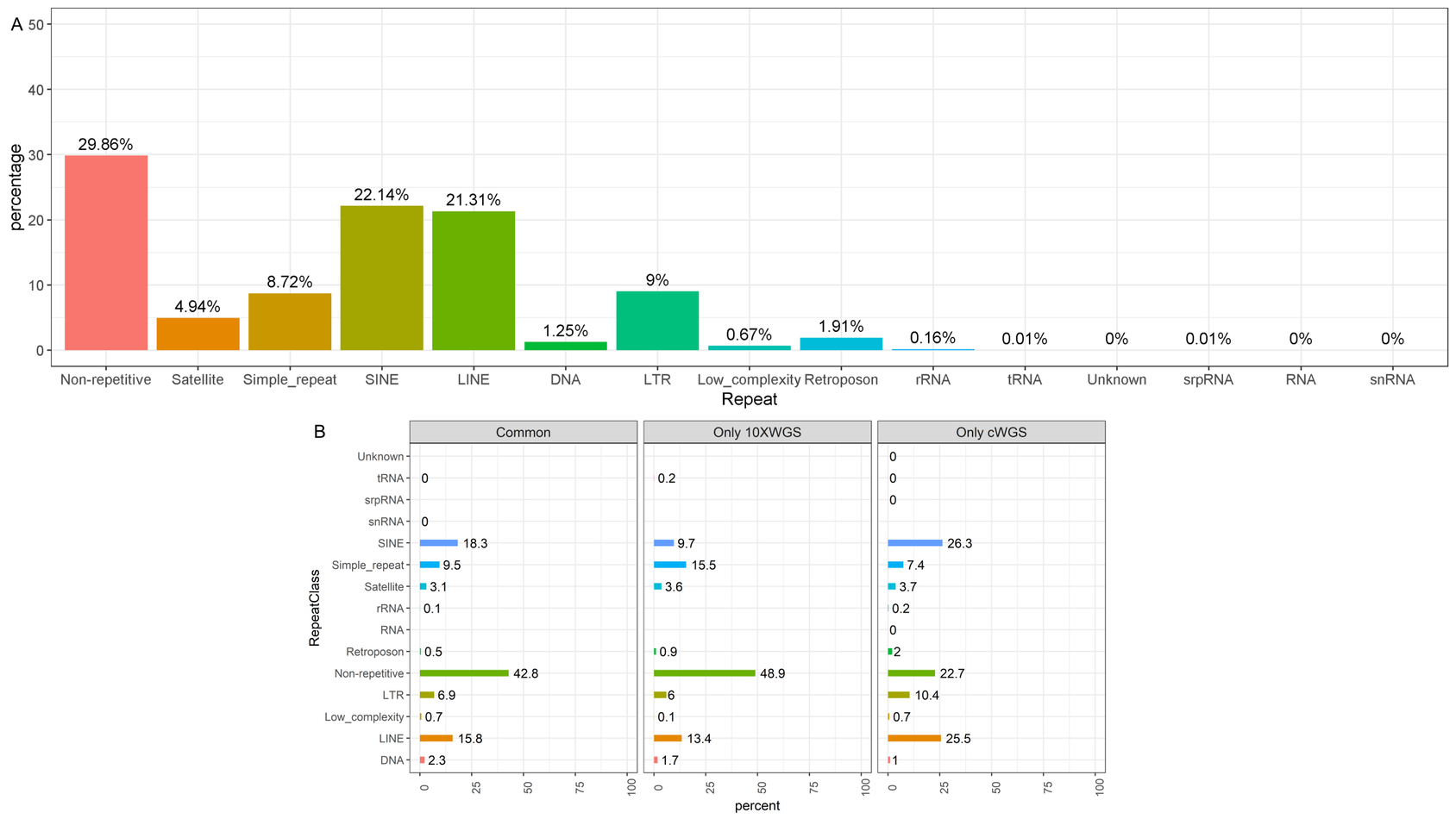

Supplement: S19 Fig — (A) The graph depicts percentage of breakpoints of SVs that lie in different repetitive regions. The SVs were filtered with the best trained combined model. (B) Breakpoints of SVs filtered by best trained combined model annotated with repetitive regions and their percentage across common, only cWGS and only 10XWGS SVs. (TIF) [file pcbi.1008397.s019.tif]

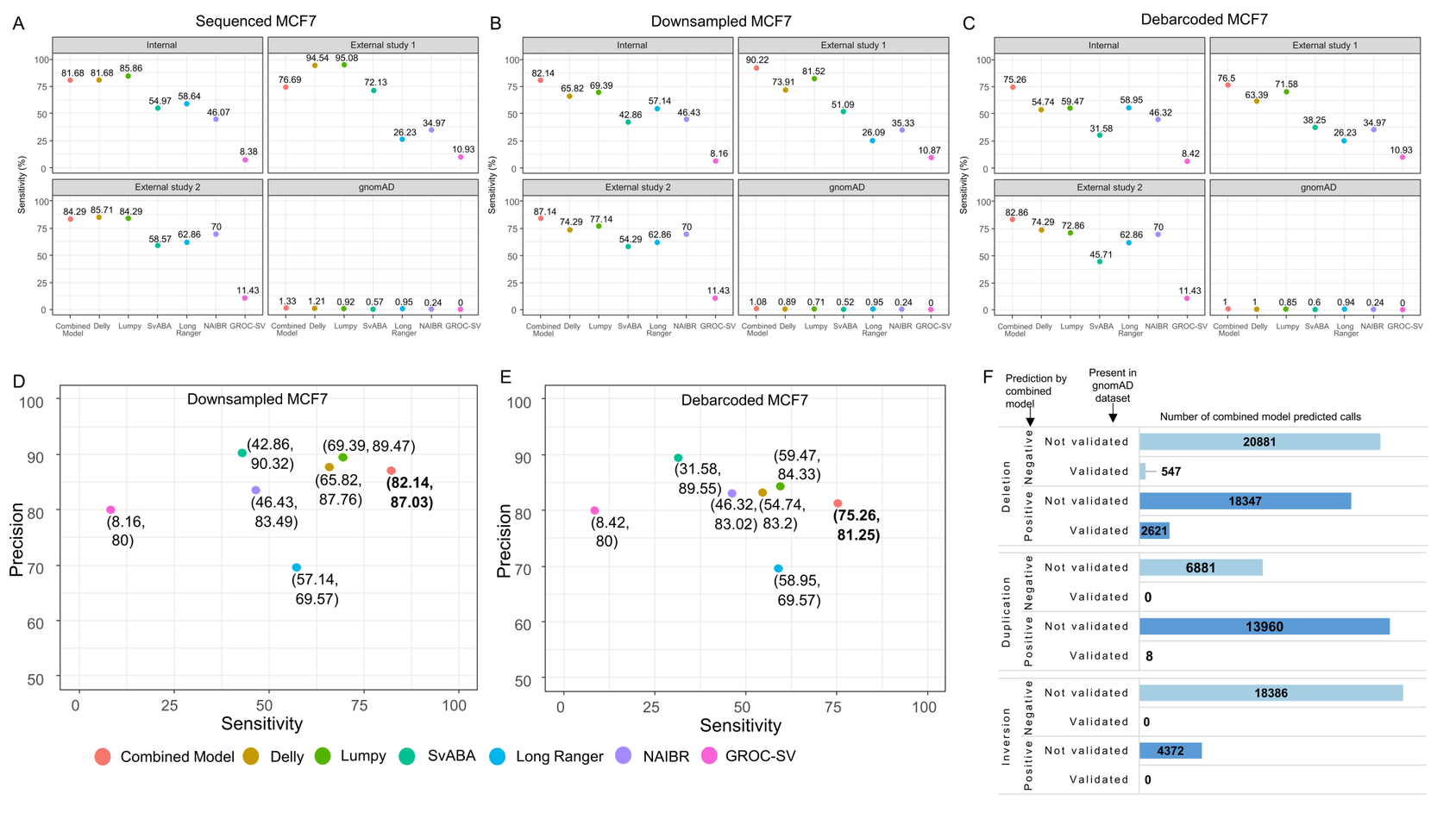

Supplement: S20 Fig — Sensitivity of combined model and other tools on the four data sets where SVs were predicted from (A) sequenced MCF7 sample. (B) downsampled cWGS MCF7 (equivalent coverage to 10XWGS MCF7 sample). (C) debarcoded 10XWGS linked-reads and processed with cWGS pipeline (for MCF7). (D) Overall performance of combined model on internally validated SVs with SVs predicted from downsampled cWGS reads. (E) Overall performance of combined model on internally validated SVs with SVs predicted from debarcoded 10XWGS linked-reads and processed with cWGS pipeline (for MCF7). (F) Number of gnomAD calls also present in SV calls filtered by the combined model in sequenced MCF7 sample. (TIF) [file pcbi.1008397.s020.tif]
